# The α9 Nicotinic Acetylcholine Receptor Mediates Nicotine-Induced PD-L1 Expression and Regulates Melanoma Cell Proliferation and Migration

**DOI:** 10.3390/cancers11121991

**Published:** 2019-12-11

**Authors:** Hai Duong Nguyen, You-Cheng Liao, Yuan-Soon Ho, Li-Ching Chen, Hui-Wen Chang, Tzu-Chun Cheng, Donald Liu, Woan-Ruoh Lee, Shing-Chuan Shen, Chih-Hsiung Wu, Shih-Hsin Tu

**Affiliations:** 1 International Master Program in Medicine, College of Medicine, Taipei Medical University, Taipei 110, Taiwan; m142106003@tmu.edu.tw; 2 Graduate Institute of Medical Sciences, College of Medicine, Taipei Medical University, Taipei 110, Taiwan; d119108004@tmu.edu.tw (Y.-C.L.); cmbwrlee@tmu.edu.tw (W.-R.L.); scshen@tmu.edu.tw (S.-C.S.); 3 TMU Research Center of Cancer Translational Medicine, Taipei Medical University, Taipei 110, Taiwan; hoyuansn@tmu.edu.tw (Y.-S.H.); d117094003@tmu.edu.tw (L.-C.C.); 4 Taipei Cancer Center, Taipei Medical University, Taipei 110, Taiwan; 5 Department of Medical Laboratory, Taipei Medical University Hospital, Taipei 110, Taiwan; g160090005@tmu.edu.tw; 6 School of Medical Laboratory Science and Biotechnology, College of Medical Science and Technology, Taipei Medical University, Taipei 110, Taiwan; d119096007@tmu.edu.tw; 7 Division of Breast Surgery, Department of Surgery, Taipei Medical University Hospital, Taipei 110, Taiwan; 8 Department of Dermatology, Taipei Medical University Shuang Ho Hospital, New Taipei City 237, Taiwan; 16308@s.tmu.edu.tw; 9 Department of Dermatology, School of Medicine, College of Medicine, Taipei Medical University, Taipei 110, Taiwan; 10 International Master/PhD Program in Medicine, College of Medicine, Taipei Medical University, Taipei 101, Taiwan; 11 Department of Surgery, EnChu Kong Hospital, New Taipei City 237, Taiwan; chwu@tmu.edu.tw; 12 Department of Surgery, School of Medicine, College of Medicine, Taipei Medical University, Taipei 110, Taiwan

**Keywords:** melanoma cells, nicotine, α9-nAChR, PD-L1, STAT3

## Abstract

Cigarette smoking is associated with an increased risk of melanoma metastasis. Smokers show higher PD-L1 expression and better responses to PD-1/PD-L1 inhibitors than nonsmokers. Here, we investigate whether nicotine, a primary constituent of tobacco, induces PD-L1 expression and promotes melanoma cell proliferation and migration, which is mediated by the α9 nicotinic acetylcholine receptor (α9-nAChR). α9-nAChR overexpression in melanoma using melanoma cell lines, human melanoma tissues, and assessment of publicly available databases. α9-nAChR expression was significantly correlated with PD-L1 expression, clinical stage, lymph node status, and overall survival (OS). Overexpressing or knocking down α9-nAChR in melanoma cells up- or downregulated PD-L1 expression, respectively, and affected melanoma cell proliferation and migration. Nicotine-induced α9-nAChR activity promoted melanoma cell proliferation through stimulation of the α9-nAChR-mediated AKT and ERK signaling pathways. In addition, nicotine-induced α9-nAchR activity promoted melanoma cell migration via activation of epithelial-mesenchymal transition (EMT). Moreover, PD-L1 expression was upregulated in melanoma cells after nicotine treatment via the transcription factor STAT3 binding to the PD-L1 promoter. These results highlight that nicotine-induced α9-nAChR activity promotes melanoma cell proliferation, migration, and PD-L1 upregulation. This study may reveal important insights into the mechanisms underlying nicotine-induced melanoma growth and metastasis through α9-nAChR-mediated carcinogenic signals and PD-L1 expression.

## 1. Introduction

Melanoma is the most aggressive and lethal form of skin cancer, accounting for fewer than 5% of all malignant skin tumors, but 75% of all skin cancer deaths in the United States [1]. Melanomas are transformed, abnormal developments of melanocytes [2]. Unlike melanocytes, melanoma cells grow uncontrollably and create a thick mass with or without ulceration [3]. As melanomas become thicker and invade deeper, melanoma cells may spread into the dermal layer, nearby tissues and other parts of the body [4]. Melanoma is divided into various histopathological subtypes, including superficial spreading, nodular, lentigo maligna, acral lentiginous, desmoplastic, muscosal and amelanotic melanoma [5]. The different subtypes of melanoma can vary substantially in their molecular characterization and pathogenesis [5]. Melanoma is the most dangerous type of skin cancer because it is the type most likely to metastasize if not diagnosed in an early stage [6]. Programmed cell death protein 1 (PD-1) blockade is the first-line therapy for advanced melanoma patients [7,8]. The mechanism of melanoma tumorigenesis is not clear and may involve a combination of environment and genetic factors [9].

Nicotinic acetylcholine receptors (nAChRs) are common ligand-activated neurotransmitter receptors located throughout the body [10]. Several subtypes of nAChRs have been demonstrated to be closely correlated to the formation and progression of tumors [10]. α9-nAChR is one of the most recently discovered nAChR subtypes [11]. At first, α9-nAChR was found in the outer hair cells of the cochlea where it is involved in auditory functions [11,12]. Subsequently, many biological functions of α9-nAChR have been identified, including keratinocyte adhesion [13], inflammation [14], chronic pain [15,16], immune responses [17], endocrine activities [18], homeostasis of osteocytes and bone mass regulation [19], and breast epithelial cancer formation [20]. α9-nAChR expression levels in breast cancer patients are strongly correlated with staging and 5-year disease-specific survival rate, and highly expresses (7.84-fold higher than the level in normal tissue) in 186 (67.3%) of the 276 breast cancer paired samples [20]. α9-nAChR short interfering RNA in MDA-MB-231 cells inhibits cell proliferation in vitro and tumor growth in vivo [20]. The α9-nAChR expression is essential for mediating tumor metastasis through epithelial-mesenchymal transition (EMT) [21,22]. Expression of EMT-related gene α9-nAChR is increased in metastasis in triple-negative breast cancer [21,22]. Notably, Chun-Yu Lin et al. reported α9-nAChR subunit could potentially correlate with 38 cancer-related pathways in 15 cancer types and interact with 64 proteins to play important roles in biological functions related to human cancer development [23]. These studies suggest that α9-nAChR may initiate signal transduction pathways, thus inducing melanoma growth and metastasis during tumor progression.

Tobacco is a known cause of cancer mortality [24]. Tobacco smoke contains 7000 toxic and carcinogenic chemicals, and various classes, such as tobacco-specific nitrosamines, polycyclic aromatic hydrocarbon, and aldehydes, are capable of inducing DNA damage response that initiates turmorigenesis and enhances metastasis [25]. Exposure to tobacco smoke is an independent risk factor for cancer [25]. The risk and severity of cancer depend on the duration of exposure and the amount of tobacco smoking [26]. In melanoma, tobacco smoking supports metastasis of melanoma cells, leading to a significant decrease in 5-year disease-free and overall survival (OS) in smokers compared to nonsmokers [27]. The correlation between current smoking and nodal metastasis of primary cutaneous melanoma is direct and positive [28]. This correlation is independent of tumor thickness, ulceration, and other risk factors [28]. An epidemiological cohort study to assess the clinical significance of α9-nAChR expression in breast tumors of different stages and to correlate α9-nAChR expression with smoking history among Taiwanese women [20]. The results revealed the increased expression of α9-nAChR mRNA in tumor tissues from current smokers relative to those from passive smokers and nonsmokers (6.62-fold vs. 2.8-fold or 1.51-fold, respectively) [20]. Nicotine is an important and common component of tobacco responsible for addiction [29]. Many studies indicate that nicotine induces breast cancer growth and metastasis in vitro and in vivo through binding to and activating α9-nAChR [20,23,30,31]. Long-term exposure to extremely low doses of nicotine and 4-(methylnitrosamino)-1-(3-pyridyl)-1-butanone (NNK) induced nonmalignant breast epithelial cell transformation through activation of the α9-nAChR-mediated signaling pathways [32]. However, the mechanisms by which nicotine-induced α9-nAChR activity promotes melanoma growth and metastasis are not fully understood.

Programmed death-ligand 1 (PD-L1) is a type 1 transmembrane protein that is encoded by the CD274 gene in humans and is overexpressed in some kinds of cancers [33]. The overexpression of PD-L1 in tumors serves as an indicator of progression and a poor prognosis [34]. In contrast, PD-L1 expression is associated with an increased response to PD-1/PD-L1 inhibitor treatment [35]. PD-1 is an inhibitory receptor that is encoded by the PDCD1 gene and is located on the surface of all T cells [35]. The PD-1 receptor on activated T cells functions in the regulation of the immune system during various physiological responses, including autoimmune disease and cancer [36]. The binding of PD-1 to PD-L1 on the surface of cancer cells leads to an immunosuppressive effect based on the interaction between phosphatases (SHP-1 or SHP-2) and an immunoreceptor tyrosine-based switch motif (ITSM) [36]. PD-L1 not only has roles in the suppression of the immune system but also has distinct tumor-intrinsic roles in growth, metastasis, and resistance to therapy [37]. Tobacco smoking-related carcinogenesis affects cancer risk by increasing the somatic mutation load [38], thereby creating neoantigens which are strongly correlated with decreased progression-free survival in cancers [39]. In contrast, neoantigens are able to stimulate immune responses and pave the way for efficacious PD-1/PD-L1 immunotherapy [40]. Several recent studies have shown positive correlations between smoking and PD-L1 expression or PD-1/PD-L1 immune checkpoint inhibitor efficacy [41]. Some clinical trials have reported that the smoking status influences OS in anti-PD-1/PD-L1-treated lung cancer patients [42]. Tobacco-smoking patients show higher PD-L1 expression [43] and better treatment responses to anti-PD-1/PD-L1 immunotherapy than patients with lung cancer who have never smoked [41]. The overall response rate to anti-PD-1/PD-L1 antibodies is lower in patients who have never smoked than in former/current smokers [41]. Recently, Wang et al. found that tobacco smoke induced PD-L1 expression in lung epithelial cells and the main tobacco carcinogens benzo(a)pyrene (BaP), dibenz[a, h]anthracence (DbA), and benzo[g, h, I]perylene (BzP) upregulated PD-L1 expression [44]. The above results suggest the possibility that nicotine may induce a mechanism that drives expression of PD-L1 in melanoma cells through α9-nAChR.

In this study, we demonstrated that α9-nAChR expression was upregulated in melanoma and associated with PD-L1 expression at the mRNA and protein levels. Overexpression of α9-nAChR in melanoma cells upregulated PD-L1 expression and enhanced melanoma cell proliferation and migration. In contrast, knocking down α9-nAChR in melanoma cells reduced PD-L1 expression and inhibited melanoma cell proliferation and migration. Nicotine-induced α9-nAChR activity upregulated PD-L1 expression and promoted melanoma cell proliferation and migration. Our results indicate novel functions for α9-nAChR in melanoma cell proliferation, migration, and PD-L1 regulation.

## 2. Results

### 2.1. α9-nAChR Is Overexpressed in Melanoma

**Figure 1:**
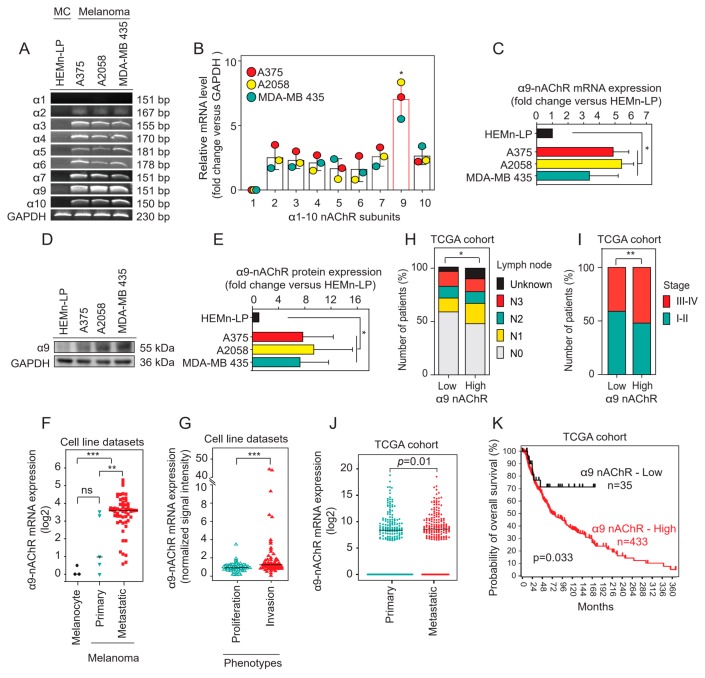
α9-nAChR expression levels and their correlations with clinicopathological parameters in multiple melanoma databases. (**A**) Detection of nAChR subunits in the primary epidermal melanocyte cell line HEMn-LP and the melanoma cell lines A375, A2058, and MDA-MB 435 by RT-PCR. (**B**) Relative mRNA expression of α1-10 nAChR subunits in the A375, A2058, and MDA-MB 435 melanoma cell lines. (**C**) Relative α9-nAChR mRNA expression in the HEMn-LP, A375, A2058, and MDA-MB 435 cell lines. (**D**,**E**) Determination of α9-nAChR mRNA levels using western blotting and statistical analysis of α9-nAChR protein levels. (**F**) The mRNA expression of α9-nAChR in two datasets from the public R2 MegaSampler platform (http://hgserver1.amc.nl/cgi-bin/r2/main.cgi) comprising melanocyte cell lines (*n* = 3) and primary (*n* = 5), and metastatic (*n* = 58) melanoma cell lines. (**G**) Screening of melanoma cell line datasets (http://www.jurno.ch/php/genehunter.html) for the mRNA expression of α9-nAChR. These cell lines were further subdivided into proliferative (*n* = 101) and invasive (*n* = 90) phenotypes. (**H**) α9-nAChR gene expression level in the TCGA-SKCM cohort (*n* = 472) downloaded from the UCSC Xena browser (https://xenabrowser.net/heatmap/). Melanoma patients were further divided into two groups based on the mean value of α9-nAChR mRNA expression, low α9-nAChR expression (*n* = 169) and high α9-nAChR expression (*n* = 291). Bar plots show the proportions of five subcategories of lymph node status in the high and low α9-nAChR level groups. (**I**) The frequencies of stages of I/II and III/IV in the high and low α9-nAChR level groups of the TCGA-SKCM cohort. (**J**) The differences in α9-nAChR expression between primary (*n* = 211) and metastatic (*n* = 201) groups. The result for the TCGA-SKCM cohort was processed using the UCSC Xena browser. (**K**) Kaplan–Meier analysis for melanoma patients based on the result from the public R2: Kaplan Meier Scanner software (https://hgserver1.amc.nl) showing a borderline difference between the groups with high (red, 433 samples) and low (black, 35 samples) α9-nAChR expression levels in the TCGA-SKCM cohort with the optimal cut-off value. (**C**,**E**) Results are shown as mean ± standard deviation (SD) of three individual experiments. *** *p* < 0.001, Student’s t-test. (**F**,**G**,**J**) The data were analyzed by the Mann-Whitney test. The median of α9-nAChR expression in each group is shown by a horizontal line. *ns*, not significant; ** *p* < 0.01; *** *p* < 0.001. (**H**,**I**) The two groups’ qualitative data were compared using the χ^2^ test; * *p* < 0.05, ** *p* < 0.01.

To evaluate the expression of nAChR subunits in three melanoma cell lines (A375, A2058, and MDA-MB 435) and a primary melanocyte cell line (HEMn-LP), we performed reverse transcriptase polymerase chain reaction (RT-PCR) to detect the mRNA levels of all alpha nAChR subunits. We found overexpression of α2-10 nAChR subunits in the A375, A2058, and MDA-MB 435 melanoma cells compared to the HEMn-LP melanocytes (Figure 1A). Quantification of the α1-10 nAChR mRNA levels in the A375, A2058, and MDA-MB 435 melanoma cells was performed based on intensities, and we found that α9-nAChR mRNA expression was more prominent than that of the other subunits of alpha nAChRs (* *p* < 0.05) (Figure 1B). α9-nAChR expression was detected in the three melanoma cell lines (A375, A2058 and MDA-MB 435) and primary melanocyte cell line (HEMn-LP) by RT-PCR (Figure 1A) and western blotting (Figure 1D and Figure S1). 

Statistical analysis found that the α9-nAChR mRNA (Figure 1C) and protein levels (Figure 1E) were obviously elevated in the three melanoma cells compared to the HEMn-LP melanocytes (* *p* < 0.05).

Melanoma cell line datasets from the public R2 MegaSampler platform (http://hgserver1.amc.nl/cgi-bin/r2/main.cgi) were evaluated. We found that α9-nAChR mRNA expression in melanoma cell lines was significantly higher than that in melanocyte cell lines (*** *p* < 0.001) (Figure 1F). In addition, α9-nAChR mRNA expression in metastatic melanoma cell lines was higher than that in primary melanoma cell lines (** *p* < 0.01) (Figure 1F).

Melanoma cell lines stratified into either a proliferative or an invasive phenotype using the melanoma cell line datasets from HOPP Database (http://www.jurmo.ch/hopp/hopp_mpse.php) were defined by a specific gene expression pattern [45]. We analyzed α9-nAChR mRNA levels and found that they were significantly upregulated in the melanoma cells (*n* = 176) with the invasive phenotype (*n* = 90) compared to those with the proliferative phenotype (*n* = 101) (*** *p* < 0.001) (Figure 1G).

We examined α9-nAChR expression of human skin cutaneous melanoma (SKCM) using the data obtained from The Cancer Genome Atlas (TCGA) from the University of California Santa Cruz (UCSC) Xena browser (https://xenabrowser.net/). The samples were divided into primary and metastatic groups according to the TNM classification for malignant melanoma staging. We found that the metastatic group had higher α9-nAChR mRNA levels than the primary group (* *p* = 0.01) (Figure 1J). Moreover, Kaplan-Meier analysis based on the result from R2: Kaplan Meier Scanner software (https://hgserver1.amc.nl) to analyze the OS of TCGA-SKMC cohort stratified according to α9-nAChR mRNA expression with an optimal cut-off value. TCGA-SKCM cohort divided into high α9-nAChR mRNA expression (433 samples) and low α9-nAChR mRNA expression (35 samples) groups (Figure 1K). 0.9 is the ratio of high α9-nAChR mRNA expression samples (433 samples) to the total amount of samples (468 samples). The results showed that the patients with high α9-nAChR expression were shorter OS than patients with low α9-nAChR mRNA expression (* *p* = 0.033) (Figure 1K).

**Figure 2:**
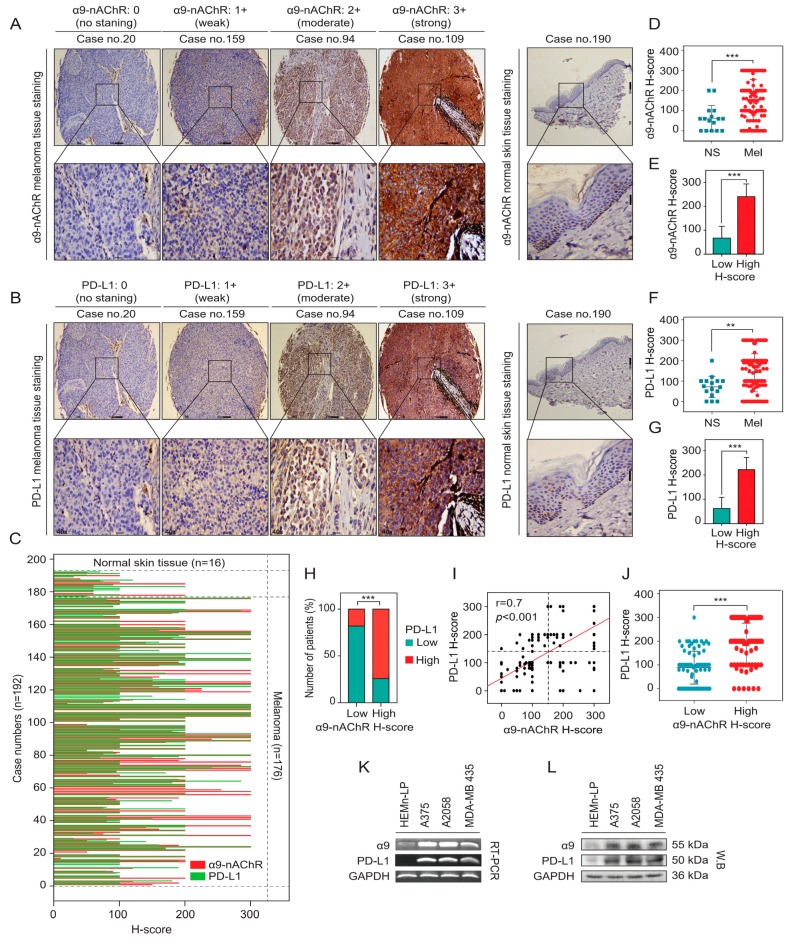
Correlations between α9-nAChR and PD-L1 in melanoma. (**A**,**B**) Representative images for α9-nAChR and PD-L1 immunohistochemistry (IHC) scoring system in the melanoma tissues and normal skin tissues. The scoring system was determined as no staining (0), weak (1+), moderate (2+), and strong (3+) based on α9-nAChR and PD-L1 intensity. (**C**) The α9-nAChR (red lines) and PD-L1 (green lines) expression profiles of melanoma tissues (*n* = 176) and normal skin tissues (*n* = 16) were detected by IHC staining based on H-score value. (**D**,**F**) Histological analysis of α9-nAChR and PD-L1 expression in melanoma tissues (Mel, *n* = 176) compared with normal skin tissues (NS, *n* = 16). (**E**,**G**) Histological analysis of α9-nAChR and PD-L1 expression in the tissue microarrays (*n* = 192) based on H-score value. The tissues were divided into two groups based on the mean value of α9-nAChR or PD-L1 H-score, low α9-nAChR (*n* = 97) or high α9-nAChR (*n* = 95) and low PD-L1 (*n* = 104) or high PD-L1 (*n* = 88). (**H**) The association between α9-nAChR and PD-L1 expression in the tissue microarrays (*n* = 192) as categorical variables. The χ^2^ test was employed to assess the correlation between a9-nAChR and PD-L1 expression in samples. *** *p* < 0.001. (**I**) Correlation between α9-nAChR and PD-L1 expression in the melanoma tissue microarrays (*n* = 192). Pearson’s rank correlation measured the strength of the association between α9-nAChR and PD-L1 expression. *** *p* < 0.001. (**J**) PD-L1 expression levels in melanoma tissues with high α9-nAChR (*n* = 89) or low α9-nAChR (*n* = 87) expression. The melanoma tissues were divided into low or high α9-nAChR based on the mean value of α9-nAChR. (**K**) α9-nAChR and PD-L1 mRNA levels detected by PT-PCR. (**L**) α9-nAChR and PD-L1 protein levels detected by western blotting. The data are presented as the mean ± SD, ** *p* < 0.01, *** *p* < 0.001, Student’s t-test.

We evaluated the immunohistochemistry (IHC) staining of α9-nAChR in tissue microarray specimens using the IHC scoring system, and the scoring system was determined as no staining (0), weak (1+), moderate (2+), and strong (3+) based on α9-nAChR intensity (Figure 2A). Histological analysis of α9-nAChR expression in melanoma tissues (Mel, *n* = 176) and normal skin tissues (NS, *n* = 16) indicated that there were significantly upregulated in melanoma tissues compared with normal skin tissues (*** *p* < 0.001) (Figure 2C,D). Taken together, these results suggested that α9-nAChR is overexpressed in melanoma and correlates with melanoma stage and phenotype.

### 2.2. Correlation of α9-nAChR Levels with Clinicopathological Features in Melanoma

To investigate the relationships between α9-nAChR and clinicopathological parameters in melanoma, we downloaded α9-nAChR gene expression data for the TCGA-SKMC cohort. We divided the patients into two groups according to the mean level of α9-nAChR. The first group expressed low levels of α9-nAChR, and the second group expressed high levels of α9-nAChR. Table 1 summarizes the associations of α9-nAChR with melanoma clinicopathological parameters (age, sex, ulceration, Breslow depth, Clark level, tumor size, lymph node status, distant metastasis status, and stage of disease). High α9-nAChR mRNA expression was significantly associated with sex (* *p* = 0.04) (Table 1), and α9-nAChR mRNA expression was significantly higher in female groups than that in male group (* *p* < 0.05) (Figure S2); however, no strong relationships were found for age, ulceration, Breslow depth or Clark level.

We next explored the correlations between α9-nAChR mRNA expression and tumor staging and observed that high α9-nAChR expression was significantly associated with lymph node metastasis (* *p* = 0.035) (Figure 1H and Table 1) and clinical stage (** *p* = 0.009) (Figure 1I and Table 1); however, no significant associations were found for tumor size or distant metastasis status in the TCGA-SKCM cohort. From these results, we suggest that α9-nAChR correlates with sex, lymph node metastasis status and clinical stage.

### 2.3. Correlation Between α9-nAChR and PD-L1 in Melanoma

We investigate whether the correlation between α9-nAChR and PD-L1 in melanoma. We analyzed the expression of α9-nAChR and PD-L1 in the melanoma tissues (*n* = 176) and normal skin tissues (*n* = 16) using the IHC staining (Figure 2A–C). The expression levels of α9-nAChR and PD-L1 were significantly increased in melanoma tissues compared with skin normal tissues (Figure 2D,F). The patient tissues were divided into two groups based on the mean value of the α9-nAChR H-score, low α9-nAChR H-score (*n* = 97) and high α9-nAChR H-score (*n* = 95) or PD-L1 H-score, low PD-L1 H-score (*n* = 104) and high PD-L1 H-score (*n* = 88). 14 cases of the normal skin tissues (*n* = 14/16) had a low α9-nAChR H-score (Figure 2C,E) and 15 cases of the normal skin tissues (*n* = 15/16) had a low PD-L1 H-score (Figure 2C,G). The chi-square (χ^2^) test was applied to assess the correlation between α9-nAChR and PD-L1 mRNA expression in samples as categorical variables (Table S1). We found that α9-nAChR expression was significantly associated with PD-L1 expression in the tissue microarrays (*** *p* < 0.001) (Figure 2H). Subsequently, we identified a strong correlation between α9-nAChR and PD-L1 expression levels in the tissue microarray data (r = 0.7, *** *p* < 0.001) (Figure 2I).

To further evaluate the correlation between α9-nAChR and PD-L1 mRNA expression, we analyzed the melanoma cell line datasets (*n* = 176) and TCGA-SKCM cohort (*n* = 472). We found that the mRNA expression of α9-nAChR was significantly associated with PD-L1 expression in the melanoma cell line datasets (*** *p* < 0.001) (Figure S3A,B) and TCGA-SKCM cohort (** *p* < 0.01) (Figure S4A,B). Furthermore, we identified a moderate correlation melanoma cell line datasets (*r* = 0.22, ** *p* = 0.003) (Figure S3C) and weak correlation between α9-nAChR and PD-L1 mRNA levels in the TCGA-SKCM cohort (*r* = 0.115, * *p* = 0.012) (Figure S4C). Melanoma is classified into various histopathological subtypes, and distinct subtypes can vary substantially in their molecular characterization and pathogenesis, which TCGA-SKCM cohort has not mentioned. Therefore, the distinct subtypes of melanoma might have different correlations between α9-nAChR and PD-L1 mRNA levels led to a weak correlation in TCGA-SKCM cohort (r = 0.115, * *p* = 0.012) (Figure 2H).

To gain more insight about the correlation of α9-nAChR and PD-L1 expression, RT-PCR and western blotting were performed in HEMn-LP, A375, A2058, and MDA-MB 435 cells using α9-nAChR and PD-L1-specific primers and antibodies, respectively. GAPDH was used as a loading control. α9-nAChR and PD-L1 expression were prominently higher in the A375, A2058, and MDA-MB 435 melanoma cells than in the HEMn-LP melanocytes at the mRNA (Figure 2K) and protein levels (Figure 2L and Figure S7). α9-nAChR expression was coupled with PD-L1 expression in the HEMn-LP, A375, A2058, and MDA-MB 435 cells at the mRNA (Figure 2K) and protein levels (Figure 2L and Figure S7). These experiments were repeated three times per cell line. Band intensities were measured using ImageJ software, and the amounts of α9-nAChR and PD-L1 at the mRNA and protein levels in the A375, A2058, and MDA-MB 435 melanoma cells were normalized to the α9-nAChR and PD-L1 mRNA and protein levels in the HEMn-LP cells. We found strong correlations between α9-nAChR and PD-L1 at the mRNA (*r* = 0.96, *** *p* < 0.001) (Figure S5A) and protein levels (*r* = 0.66, * *p* = 0.02) (Figure S5B) in the HEMn-LP, A375, A2058, and MDA-MB 435 cells.

On the other hand, we compared PD-L1 expression levels between the low α9-nAChR (melanoma tissue microarrays, *n* = 87; TCGA-SKCM cohort, *n* = 167; melanoma cell line datasets, *n* = 156) and high α9-nAChR (melanoma tissue microarrays, *n* = 89; TCGA-SKCM cohort, *n* = 305; melanoma cell line datasets, *n* = 20) expression groups and found that PD-L1 expression levels were significantly higher in the samples with high α9-nAChR expression than in the samples with low α9-nAChR expression (** *p* < 0.01, *** *p* < 0.001) (Figure 2J, Figures S3D and S4D). According to the results, we suggest that α9-nAChR expression correlates with the PD-L1 expression in melanoma.

### 2.4. α9-nAChR Induces PD-L1 Expression and Regulates in Proliferation and Migration

**Figure 3:**
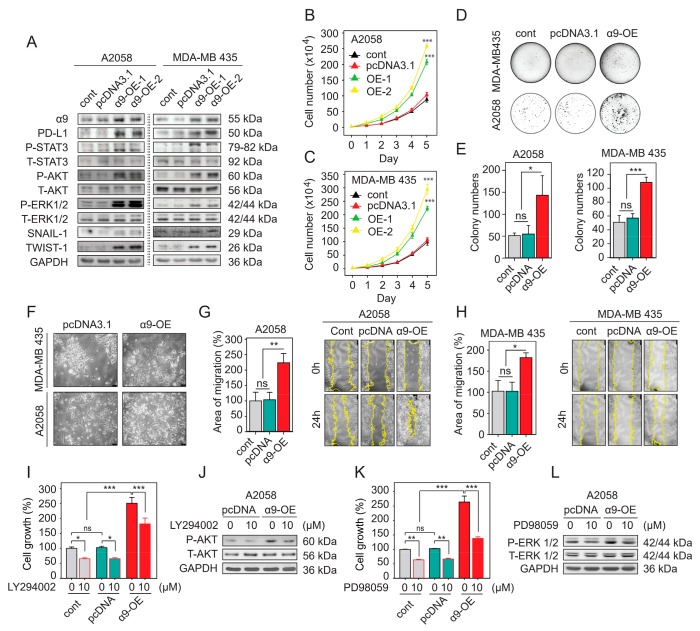
α9-nAChR overexpression induces PD-L1 upregulation and promotes melanoma cell proliferation and migration. (**A**) Overexpression of α9-nAChR activated the phosphorylation of AKT, ERK, and STAT3 and induced PD-L1, Snail-1, and Twist-1 protein levels in A2058 and MDA-MB-435 cells, which were evaluated by western blotting. (**B**,**C**) α9-nAChR overexpression significantly increased the proliferation of A2058 and MDA-MB435 cells compared to control cells, as determined by a cell count assay. (**D**) Soft-agar growth assays were performed with A2058 and MDA-MB-435 cells transfected with pcDNA3.1 or pcDNA3.1-α9-nAChR overexpression vectors. (**E**) Colony numbers were quantified and presented in the histogram. (**F**) Cell morphology of A2058 and MDA-MD-435 cells transfected with pcDNA3.1 or pcDNA3.1-α9-nAChR overexpression vector. (**G**,**H**) Representative micrographs and statistical analysis demonstrated that α9-nAChR overexpression increased the migration areas of A2058 and MDA-MB 435 cells at 24 h after scratching. (**I**,**K**) Percentages of cell proliferation for the A2058 cells, pcDNA3.1-A2058 cells, and α9-nAChR-overexpressing A2058 cells were treated with or without the PI3K inhibitor LY294002 (10 µm) or MEK inhibitor PD98059 (10 µm) for 48 h determined by an MTT assay. (**J**,**L**) Western blotting to detect the protein levels of P-AKT and P-ERK in pcDNA3.1-A2058 cells and α9-nAChR-overexpressing A2058 cells with or without LY294002 or PD98059 treatment. The data are presented as the mean ± SD of three independent experiments. * *p* < 0.05, ** *p* < 0.01, *** *p* < 0.001, Student’s t-test.

**Figure 4:**
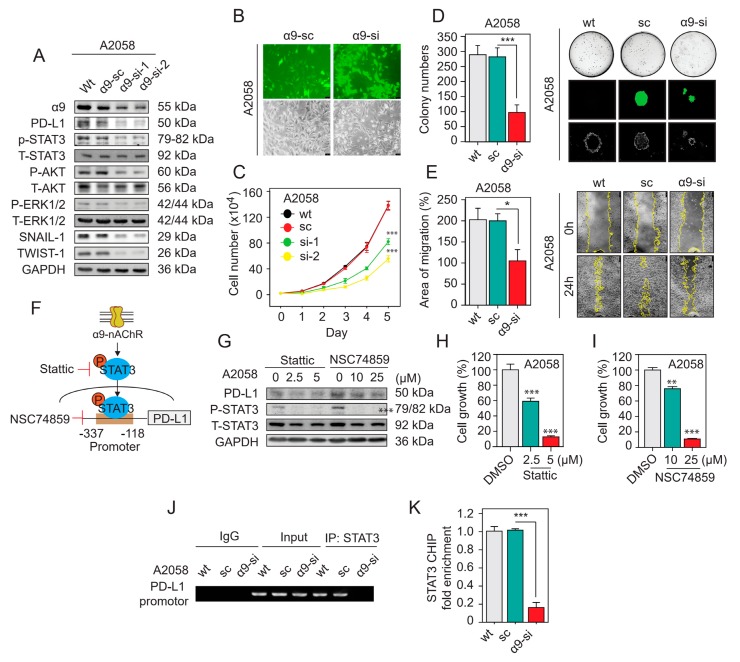
Knocking down α9-nAChR expression inhibits melanoma cell proliferation and migration and affects the protein level of PD-L1. (**A**) Western blotting results showing the protein level changes in α9-nAChR, PD-L1, P-STAT3, P-AKT, P-ERK, Snail-1 and Twist-1 after knocking down α9-nAChR expression in A2058 cells. GAPDH served as a control. (**B**) Fluorescence micrographs showing GFP expression (bottom panels) of α9-nAChR-siRNA-expressing or scrambled siRNA-A2058 cells at 48 h after pSUPER-α9-nAChR-si or pSUPER-α9-nAChR-scramble vector transfection, and corresponding phase-contrast microscopy images showing cell morphology (top panels). (**C**) Cell proliferation of α9-nAChR-siRNA-expressing A2058 cells monitored by a cell count assay. (**D**) Cell proliferation of α9-siRNA-expressing A2058 cells assessed by a soft-agar growth assay. The colonies formed in the low density seeding assay were counted after 21 days (left panels). The amounts, sizes, and fluorescence microscopy images of colonies are shown (right panels). (**E**) Migratory capacity measured at 24 h using a wound-healing assay with A2058 cells stably expressing α9-nAChR-siRNA (si), scrambled siRNA (sc) or wild-type (wt) (left panels). Quantification of the migratory areas (right panels) were measured using ImageJ software. (**F**) The STAT3 binding site of the PD-L1 promoter and targets of STAT3 phosphorylation inhibitor Stattic and STAT3 DNA-binding inhibitor NSC74859. (**G**) Effects of treatment with the inhibitors Stattic and NSC74858 on P-STAT3, and PD-L1 protein levels in A2058 cells evaluated by western blotting. GAPDH served as a control. (**H**,**I**) Inhibitors Stattic and NSC74859 inhibited A2058 cell proliferation. (**J**,**K**) ChIP assay using STAT3-precipitated DNA samples from wild-type, scrambled siRNA, and α9-nAChR-siRNA-expressing A2058 cells. The DNA fragments were subjected to RT-PCR and real-time PCR (qPCR) using PD-L1 promoter primer pairs as shown in Table S2. In RT-PCR, a rabbit IgG antibody was used as a negative control, and total genomic DNA was used as a positive control. In qPCR, relative fold enrichments of PD-L1 promoter region were calculated after normalization to GAPDH. Each of the immunoprecipitations was replicated three times, and each sample was quantified at least in triplicate. The data are presented as the mean ± SD of independent experiments. * *p* < 0.05, ** *p* < 0.01, *** *p* < 0.001, Student’s t-test.

The results for the melanoma databases, cell lines, and tissue microarrays indicated that α9-nAChR was highly associated with tumor growth, metastasis, and PD-L1 expression in melanoma patients and with cell proliferation, migration, and PD-L1 expression in melanoma cell lines. To evaluate the roles of α9-nAChR in the proliferation, migration and PD-L1 upregulation of melanoma cells, we generated stable α9-nAChR-siRNA-expressing A2058 cells, α9-nAChR-overexpressing A2058 cells and α9-nAChR-overexpressing MDA-MB 435 cells by transfection with α9-nAChR-small interfering RNA (siRNA) or pcDNA3.1-α9-nAChR overexpression vectors. Stable scrambled siRNA-A2058 cells, pcDNA3.1-A2058 cells, and pcDNA3.1-MDA-MB 435 cells were used as control groups. The protein levels of α9-nAChR expression in the cells with α9-nAChR knockdown or overexpression were compared to the protein levels in the control cells (Figure 3A and Figure 4A, Figures S8 and S10). α9-nAChR overexpression significantly increased cell proliferation in the A2058 (Figure 3B) and MDA-MB 435 cells (Figure 3C) after five days incubation by a cell count assay (*** *p* < 0.001). These results were confirmed by a soft-agar colony assay at 21 days (*ns*, not significant; * *p* < 0.05; *** *p* < 0.001) (Figure 3D,E). In contrast, compared to stable scrambled siRNA-A2058 cells, stable α9-nAChR-siRNA-expressing A2058 cells substantially decreased the numbers of cells and colonies after 5 and 21 days, respectively (*** *p* < 0.001) (Figure 4C,D). Together, these data demonstrate that α9-nAChR regulates melanoma cell proliferation in vitro.

We performed a scratch-wound healing assay to examine whether α9-nAChR affects the migration of melanoma cells. After scratching, cells along the wound-edge migrated into the empty space continuously so that the migration area, as indicated by comparing the distance between the opposite cell edges at 0 h and 24 h, became wider over time. We compared stable α9-nAChR-overexpressing A2058 cells to stable pcDNA3.1-A2058 cells and showed apparent increases in the migration areas at 24 h after scratching (*ns*, not significant; ** *p* < 0.01) (Figure 3G). For stable α9-nAChR-overexpressing MDA-MB 435 cells, the migration areas were also significantly larger than those of pcDNA3.1-MDA-MB 435 cells at 24 h after scratching (*ns*, not significant; * *p* < 0.05) (Figure 3H). In contrast, compared to corresponding stable scrambled siRNA-A2058 cells, stable α9-nAChR-siRNA-expressing A2058 cells exhibited substantially reduced migration areas at 24 h after scratching (Figure 4E, right panel). Statistical analysis demonstrated that the migration areas of the stable α9-nAChR-siRNA-expressing A2058 cells were significantly narrower than those of the stable scrambled siRNA-A2058 cells at 24 h after scratching (* *p* < 0.05) (Figure 4E, left panel). Taken together, our results demonstrate that α9-nAChR positively regulates melanoma cell migration.

α9-nAChR expression correlated with PD-L1 expression in melanoma (Figure 2 and Figures S3–S5). To further understand the relationship between α9-nAChR and PD-L1 in melanoma cells, we performed western blotting to assess PD-L1 protein levels in stable α9-nAChR-knockdown and α9-nAChR-overexpressing cells. We found that the protein level of PD-L1 was significantly upregulated in stable α9-nAChR-overexpressing A2058 and MDA-MB 435 cells compared to the corresponding stable pcDNA3.1-expressing A2058 and MDA-MB 435 cells (Figure 3A and Figure S8). On the other hand, the protein level of PD-L1 was substantially downregulated in the stable α9-nAChR-siRNA-expressing A2058 cells compared to the stable scrambled siRNA-A2058 cells (Figure 4A and Figure S10). According to these results, we suggest that α9-nAChR induces melanoma cell proliferation, migration, and PD-L1 upregulation.

### 2.5. α9-nAChR Regulates Melanoma Cell Proliferation via the AKT and ERK Signaling Pathways

To investigate the mechanisms by which α9-nAChR regulates proliferation, western blot analysis was performed with melanoma cells to detect the protein levels of AKT and ERK, which are well-documented signaling molecules associated with tumor growth [2]. We found that α9-nAChR affected the phosphorylated forms of AKT and ERK1/2 in α9-nAChR-siRNA-expressing A2058 cells, α9-nAChR-overexpressing A2058 cells and α9-nAChR-overexpressing MDA-MB 435 cells (Figure 3A and Figure 4A, Figures S8 and S10). As shown in Figure 3A and Figure S8, overexpression of α9-nAChR in A2058 cells and MDA-MB 435 cells substantially promoted the activation of the AKT and ERK signaling pathways. In contrast, α9-nAChR depletion in A2058 cells significantly decreased AKT and ERK activation (Figure 4A and Figure S10).

To elucidate the role of α9-nAChR in promoting melanoma cell proliferation via the AKT and ERK signaling pathways, we examined the proliferative ability of stable α9-nAChR-overexpressing A2058 cells treated with the PIK3 inhibitor LY 294002 (10 μm) or MEK inhibitor PD 98059 (10 μm) for 48 h and compared the proliferation of these cells to that of stable pcDNA3.1-A2058 cells treated with the inhibitors. Western blotting was performed to evaluate the inhibitory control of PIK3 inhibitor LY 294002 or MEK inhibitor PD 98059 on the phosphorylation of AKT and ERK (Figure 3J,L and Figure S9). We found that an inhibitory effect on the proliferation of the A2058 cells when α9-nAChR overexpression was combined with the PI3K or MEK inhibitor (*** *p* < 0.001) (Figure 3I,K). Together, these data suggest that α9-nAChR plays a role in the proliferation of melanoma cells through the activation of the AKT and ERK signaling pathways.

### 2.6. α9-nAChR Promotes Melanoma Cell Migration through EMT

The fact that α9-nAChR overexpression/knockdown greatly affected melanoma cell movement strongly suggested a major function for α9-nAChR in controlling melanoma cell migration. To elucidate the underlying mechanism, we observed morphological changes in cells with α9-nAChR overexpression or knockdown compared to cells transfected with control vectors. The results demonstrated that α9-nAChR overexpression in A2058 and MDA-MB 435 cells induced mesenchymal-like melanoma cells with loss of cell-cell adhesions junctions (Figure 3F). Conversely, α9-nAChR knockdown in A2058 cells resulted in increased cell-cell adhesions junctions and epithelial-like melanoma cells (Figure 4B). The molecular changes in the levels of EMT markers (Twist-1 and Snail-1) which are pivotal in controlling cancer cell migration via cell transformation [46], also indicated the occurrence of EMT in cells with α9-nAChR overexpression/knockdown. Moreover, it is known that MEK and PI3K cascades promote EMT by upregulating Twist-1 and Snail-1 expression [46]. The western blot analysis clearly showed that compared to stable scrambled siRNA-A2058 cells, stable α9-nAChR-siRNA-expressing A2058 cells inhibited the activation of AKT and EKR phosphorylation and significantly downregulated the expression of the EMT markers Twist-1 and Snail-1 (Figure 4A and Figure S10). In contrast, compared to pcDNA3.1-A2058 cells and pcDNA3.1-MDA-MB 435 cells, α9-nAChR overexpressing A2058 cells and α9-nAChR overexpressing MDA-MB 435 cells exhibited activated MEK and PI3K signaling pathways and substantially increased expression of EMT markers, including Twist-1 and Snail-1, at the protein level (Figure 3A and Figure S8). Based on these results, we suggest that α9-nAChR regulates melanoma cell migration through EMT.

### 2.7. α9-nAChR Regulates PD-L1 Expression via the STAT3 Signaling Pathway

We investigate whether the STAT3 signaling pathway regulates PD-L1 expression. We treated A2058 cells with the STAT3 phosphorylation inhibitor Stattic (2.5 μm or 5 μm) and STAT3 DNA-binding inhibitor NSC74859 (10 μm or 25 μm) to inhibit STAT3 activation (Figure 4F). Western blotting and MTT assay were performed to evaluate the PD-L1 expression and cell growth, respectively. Stattic and NSC74859 significantly inhibited STAT3 activation, leading to the suppression of PD-L1 expression (Figure 4G and Figure S11) and cell growth (Figure 4H,I) in A2058 cells. From this evidence, we conclude that STAT3 signaling pathway regulates cell growth and PD-L1 expression in melanoma cells.

We examined the role of the α9-nAChR-mediated STAT3 signaling pathway in regulating PD-L1 expression. We found that α9-nAChR overexpression in A2058 and MDA-MB 435 cells substantially increased phosphorylated STAT3 expression (Figure 3A and Figure S8). In contrast, knocking down α9-nAChR in A2058 cells significantly decreased phosphorylated STAT3 expression (Figure 4A and Figure S10).

Several recent findings have mentioned that -337 to -118 fragment of the PD-L1 core promoter is enriched with consensus binding sites for STAT3, which is known to regulate PD-L1 expression transcriptionally (Figure 4F) [47,48]. To investigate the mechanism involving the transcription factor STAT3 in α9-nAChR-mediated PD-L1 expression, we performed a chromatin immunoprecipitation (ChIP) assay with an anti-STAT3 rabbit polyclonal antibody and primer pairs specific for the PD-L1 gene promoter to compare stable α9-nAChR-siRNA-expressing A2058 cells with stable scrambled siRNA-A2058 cells. The expression of PD-L1 was evaluated by RT-PCR and qPCR. We found that the transcription factor STAT3 directly bound to the promoter of PD-LP at -337 to -118 region (Figure 4J). Moreover, knocking down α9-nAChR expression decreased the PD-L1 level by reducing STAT3 binding to the PD-L1 promoter (*** *p* < 0.001) (Figure 4K). Thus, we suggest that PD-L1 expression is directly regulated by the STAT3 downstream signaling transduction pathway activated by α9-nAChR.

### 2.8. Nicotine-Induced α9-nAChR Activity Upregulates PD-L1 Expression and Promotes Melanoma Cell Proliferation and Migration

**Figure 5:**
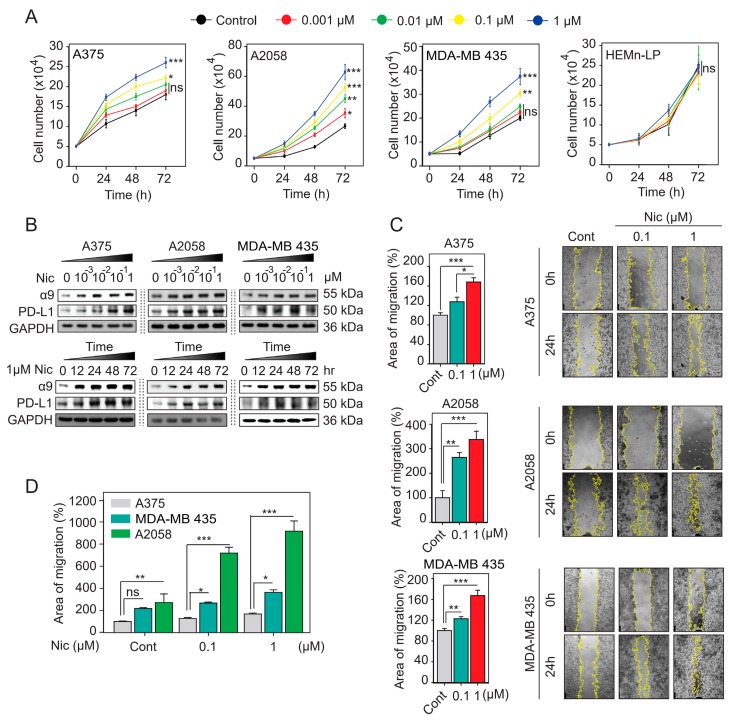
Nicotine-induced α9-nAChR activity promotes melanoma cell proliferation, migration, and PD-L1 upregulation. (**A**) Nicotine induced melanoma cell proliferation. Cell count assays were performed with the A375, A2058, and MDA-MB 435 melanoma cells and HEMn-LP melanocytes, and the cells were treated with nicotine at the final concentrations of 0.001–1 μm for 24–72 h. (**B**) Melanoma cells were exposed to nicotine at the indicated concentrations for indicated time periods. α9-nAChR and PD-L1 protein levels were determined by western blotting. (**C**) Nicotine promoted melanoma cell migration. Cell migration capabilities were evaluated using a wound-healing assay. Cells were scratched using 10 or 100 μL tips and treated with nicotine at the indicated concentrations for 24 h. (**D**) Comparing migration area among the A375, A2058, and MDA-MB 435 melanoma cells. The data are presented as the mean ± SD of three independent experiments. ns, not significant; * *p* < 0.05; ** *p* < 0.01; *** *p* < 0.001; Student’s t-test.

**Figure 6:**
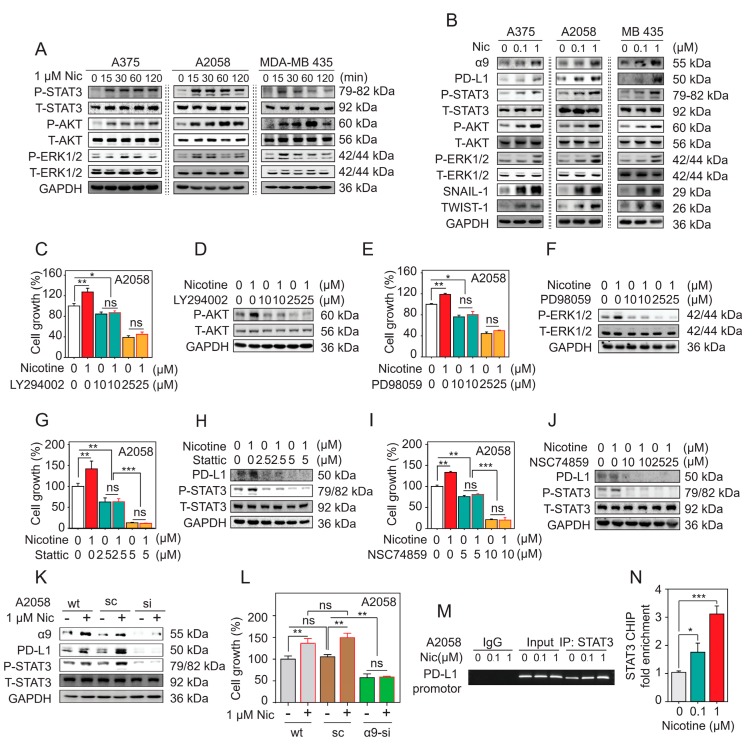
α9-nAChR mediates nicotine-induced PD-L1 expression and regulates melanoma cell proliferation. (**A**) Nicotine increased STAT3, AKT, and ERK phosphorylation in a time-dependent manner in the A375, A2058, and MDA-MB-435 cells, as assessed by western blotting. (**B**) Nicotine induced the phosphorylation of STAT3, AKT, and ERK and upregulated the protein levels of α9-nAChR, PD-L1, Snail-1 and Twist-1 in a dose-dependent manner. The results were determined by western blotting. (**C**–**J**) A2058 cells were pretreated with the inhibitor LY294002 (10 μm or 25 μm), PD98059 (10 μm or 25 μm), Stattic (2.5 μm or 5 μm) or NSC74859 (10 μm or 25 μm) for 3-24 h and subsequently treated with or without nicotine (1 μm) for additional 24-48 h. Western blot analysis was used to detect protein levels. Cell proliferation was assessed by an MTT assay. (**K**,**L**) Wild-type, scrambled siRNA, and α9-nAChR-si-expressing A2058 cells were treated with or without 1 μm nicotine. Western blot analysis was used to assess protein levels. Cell growth was assessed by an MTT assay. (**M**,**N**) After A2058 cells were exposed to 0.1 μm or 1 μm nicotine for 24 h, a ChIP assay was performed using an anti-STAT3 antibody. The protein-chromatin immunoprecipitates were subjected to RT-PCR and qPCR. In RT-PCR, a rabbit IgG antibody was used as a negative control, and total genomic DNA was used as a positive control. In qPCR, relative enrichments of PD-L1 promoter region were calculated after normalization to GAPDH. Each of the immunoprecipitations was replicated three times, and each sample was quantified at least in triplicate. The data are presented as the mean ± SD of three independent experiments. ns, not significant; * *p* < 0.05; ** *p* < 0.01; *** *p* < 0.001; Student’s t-test.

We examined the effects of nicotine-induced α9-nAChR activity on cell proliferation in the HEMn-LP melanocytes and A375, A2058, and MDA-MB 435 melanoma cells using nicotine at final concentrations of 0.001–1 μm to treat the cells for 24–72 h. As shown in Figure 5A,B and Figure S12, we found that nicotine-induced α9-nAChR activity significantly promoted cell proliferation in a concentration and time-dependent manner in the three melanoma cell lines (A375, A2058, and MDA-MB 435), with maximal proliferation occurring with the 1 μm dose (ns, not significant; * *p* < 0.05; ** *p* < 0.01; *** *p* < 0.001). However, we found no significant effect of nicotine-induced α9-nAChR activity on HEMn-LP melanocyte growth (ns, not significant) (Figure 5A, Figures S6 and S18). Compared with that in stable scrambled siRNA-A2058 cells, α9-nAChR-siRNA-expressing A2058 cells showed an inhibition of nicotine-stimulated proliferation (Figure 6K,L and Figure S17). These results suggest that nicotine-induced α9-nAChR activity promotes melanoma cell proliferation.

To examine the effects of nicotine on migration in the A375, A2058, and MDA-MB 435 melanoma cells, we performed a scratch-wound healing assay. After scratching, the cells were treated with nicotine at a final concentration of 0.1 or 1 μm for 24 h. By comparing migration areas at 0 h and 24 h, we found that the migration areas were significantly larger in the nicotine treatment groups than in the groups without nicotine treatment after 24 h (* *p* < 0.05, ** *p* < 0.01, *** *p* < 0.001) (Figure 5C). When comparing migration area among the three cell lines, the migration areas of the A2058 cells were the largest (*ns*, not significant; * *p* < 0.05; ** *p* < 0.01; *** *p* < 0.001) (Figure 5D). Moreover, exposure of all three cell lines to nicotine induced increases in Twist-1 and Snail-1 protein levels (Figure 6B and Figure S14). These results suggest that exposure to nicotine induces EMT and promotes cell migration through α9-nAChR in the A375, A2058, and MDA-MB 435 melanoma cells. To investigate nicotine-induced α9-nAChR and PD-L1 upregulation, we performed western blotting to detect α9-nAChR and PD-L1 protein levels in the A375, A2058 and MDA-MB 435 melanoma cells treated with nicotine at final concentrations of 0.001–1 μm for 24 h or at a concentration of 1 μm for 12–72 h. We found that nicotine induced both α9-nAChR and PD-L1 upregulation in a concentration- and time-dependent manner (Figure 5B and Figure S12). However, in the HEMn-LP melanocytes, nicotine-induced α9-nAChR activity did not upregulate PD-L1 expression (Figures S6 and S18).

Wild-type, scrambled siRNA, and α9-nAChR-siRNA-expressing A2058 cells were treated with or without 1 μm nicotine. After 24 h, we compared α9-nAChR-siRNA-expressing A2058 cells treated with 1 μm nicotine to those not treat with 1 μm nicotine. We found that knocking down α9-nAChR led to an inhibition of nicotine-induced PD-L1 protein levels through α9-nAChR-mediated STAT3 signaling pathway (Figure 6K and Figure S17). Together, these results suggest that α9-nAChR mediates nicotine-induced PD-L1 expression.

### 2.9. Nicotine-Induced α9-nAChR Activity Significantly Increases Melanoma Cell Proliferation via the AKT and ERK Signaling Pathways

To examine the mechanisms by which the AKT and ERK signaling pathways induced nicotine-stimulated α9-nAChR activity promote proliferation in the A375, A2058, and MDA-MB 435 melanoma cells, we treated cells with 1 μm nicotine at the indicated time points (Figure 6A and Figure S13). Nicotine increased AKT and ERK phosphorylation in a time-dependent manner in the A375, A2058 and MDA-MB 435 melanoma cells, as assessed by immunoblotting with anti–phospho-AKT and anti–phospho-ERK antibodies (Figure 6A and Figure S13). To evaluate concentration-dependent responses to nicotine, we treated cells with nicotine at final concentrations of 0.1–1 μm for 24 h. We found that nicotine increased the phosphorylation of AKT and ERK level in the three cell lines in a concentration-dependent manner (Figure 6B and Figure S14).

To examine the potential roles of PI3K and MEK inhibitors in the prevention of nicotine-induced melanoma cell proliferation, we pretreated A2058 cells with LY 294002 (10 μm and 25 μm) or PD 98059 (10 μm and 25 μm) for 3 h and then treated the cells with or without 1 μm nicotine for 24–48 h. As shown in Figure 6C–F and Figure S15, LY294002 and PD98059 significantly inhibited the activation of the AKT and ERK signaling pathways, leading to the inhibition of nicotine-induced melanoma cell proliferation. Moreover, knocking down α9-nAChR expression led to an inhibition of the nicotine-induced proliferation mediated by the AKT and ERK signaling pathways (Figure 6L). Together, these data indicate that nicotine-induced α9-nAChR activity significantly increases melanoma cell proliferation via the AKT and ERK signaling pathways.

### 2.10. Nicotine-Induced α9-nAChR Activity Upregulates PD-L1 Expression via STAT3 Signaling Pathway

The transcription factor STAT3 is required for regulating PD-L1 expression. To examine whether nicotine-induced α9-nAChR activity activated the transcription factor STAT3 in response to PD-L1 upregulation, we performed western blotting and ChIP assays. Nicotine induced STAT3 phosphorylation and upregulated PD-L1 protein levels in a time- and dose-dependent manner (Figure 5B and Figure 6A,B, Figures S12–S14)). Pretreatment of A2058 cells with the STAT3 phosphorylation inhibitor Stattic (2.5 μm or 5 μm) or STAT3 DNA-binding inhibitor NSC74859 (10 μm or 25 μm) to block the activation of the STAT3 signaling pathway was performed for 24 h prior to a coincubation with or without 1 μm nicotine stimulation. As shown in Figure 6H,J and Figure S16, the inhibitors Stattic and NSC74859 significantly inhibited the stimulation of the nicotine-activated STAT3 signaling pathway that leads to PD-L1 upregulation. Moreover, the inhibitors Stattic and NSC74859 prevented the nicotine-induced melanoma cell proliferation (Figure 6G,I). Knocking down α9-nAChR expression led to an inhibition of the nicotine-induced PD-L1 protein level through the STAT3 signaling pathway (Figure 6K and Figure S17). We added 0.1 or 1 μm nicotine to A2058 cells for 24 h, and then performed a ChIP assay with an anti-STAT3 antibody and primer pairs specific for the PD-L1 gene promoter. The expression of PD-L1 was evaluated by RT-PCR and qPCR. We found that nicotine activated the transcription factor STAT3, which directly bound to the promoter of PD-L1 in -337 to -118 region (Figure 6M). Moreover, nicotine upregulated PD-L1 levels by increasing STAT3 binding to the PD-L1 promoter in a dose-dependent manner (* *p* < 0.05, *** *p* < 0.001) (Figure 6N). Together, these results indicate that nicotine-induced α9-nAChR activity activates the STAT3 signaling pathway that influences melanoma cell proliferation and upregulates PD-L1 expression, and the STAT3 inhibitors Stattic and NSC74859 prevent nicotine-induced PD-L1 expression and proliferation.

## 3. Discussion

Smoking is the leading cause of premature death and causes so many types of cancers [49]. Increasing evidence indicates that nicotine, which is one of the harshest substances in tobacco smoke, contributes to carcinogenesis in various cancer types, affects cancer progression and results in a poor prognosis in patients [30]. Nicotine binds to nAChRs and induces various downstream signaling pathways involved in cancer promotion, progression, and malignancy [30]. Recently, various epidemiological studies reported that tobacco smoking significantly increases metastasis in melanoma [27,28]. Moreover, a previous study indicated that α9-nAChR had important roles in promoting breast cancer growth and metastasis in vitro and in vivo [20]. In our results, we showed, for the first time, the associations of α9-nAChR expression with clinicopathological features of melanoma patients, including clinical staging, lymph node status and OS (Figure 1 and Table 1). α9-nAChR expression was significantly higher in melanoma cells than in melanocytes based on four cell lines (A375, A2058, and MDA-MB 435 melanoma cell lines and HEMN-LP melanocyte cell line) and melanoma cell line datasets (Figure 1). α9-nAChR mRNA level was higher in melanoma cells with the invasive phenotype than in those with the proliferative phenotype (Figure 1). In the TCGA-SKCM cohort, the metastatic patient group had higher α9-nAChR mRNA level than the primary patient group. In vitro, overexpression of α9-nAChR via transfection substantially increased melanoma cell proliferation and migration (Figure 3). By contrast, the suppression of α9-nAChR expression significantly inhibited melanoma cell growth and migration (Figure 4). Taken together, these findings suggest that α9-nAChR expression is not only relevant to melanoma growth and metastasis but also an unfavorable prognostic factor in melanoma patients.

α9-nAChR mRNA expression significantly associated with sex (* *p* = 0.04) (Table 1), and female had a higher α9-nAChR mRNA expression as compared to male (* *p* < 0.05) (Figure S2). 17β-estradiol upregulated α9-nAChR expression via the phosphorylation of estrogen receptor-alpha (ERα)-mediated the binding of AP1 and transcription factors to the α9-nAChR gene promoter, and –induced α9-nAChR protein production in breast cancer cells [31]. This study may provide us the clues to explain the mechanism of female sex hormone-induced α9-nAChR expression.

Numerous findings indicate that PD-L1 is present in tumor cells [50]. Tumor-expressed PD-L1 binds to PD-1-expressed on T cells, leading to the alterations of tumor immunopathogenesis which helps tumor cells evade the immune response [50]. Furthermore, the roles for tumor-intrinsic-PD-L1 in the regulation of EMT, cancer stemness, tumor development, metastasis, and resistance to therapy have recently been discovered [37]. PD-L1 overexpression, knockdown or knockout in glioblastoma multiforme was investigated, and the results demonstrated that PD-L1 promoted cancer cell growth and metastasis in vitro and in vivo [51]. In our study, the expression of PD-L1 is overexpressed in melanoma cells and tissues compared with melanocytes and normal skin tissues, respectively (Figure 2); and the positive correlation between α9-nAChR and PD-L1 expression by using melanoma cell lines, tissue microarrays, and public databases (Figure 2 and Figures S3–S5). In particular, α9-nAChR knockdown or overexpression induced changes in PD-L1 expression (Figure 3 and Figure 4). We suggest that α9-nAChR promotes melanoma cell proliferation and migration through the regulation of PD-L1.

The transcription factor STAT3 plays a key role in the regulation of PD-L1 expression in various cancer types [52]. In non-Hodgkin lymphoma, STAT3 binds to the PD-L1 gene promoter, resulting in upregulation of PD-L1 expression in vitro and in vivo [53]. STAT3 suppression decreases PD-L1 expression in ALK-positive anaplastic large cell lymphoma (ALCL) [54] and KRAS-mutant non-small cell lung cancer (NSCLC) cells [55]. In our study, STAT3 was activated and regulated by α9-nAChR (Figure 3 and Figure 4). Moreover, we found that STAT3 bound to the PD-L1 promoter in melanoma cells, and knocking down α9-nAChR expression inhibited STAT3 binding to the PD-L1 promoter, leading to PD-L1 downregulation (Figure 4). In contrast, the stimulation of α9-nAChR expression with nicotine increases STAT3 binding to PD-L1 promoter, leading to PD-L1 upregulation (Figure 6).

α9-nAChR synergizes mainly with epidermal growth factor (EGF) receptor, insulin-like growth factor 1 (IGF-1) receptor, vascular endothelial growth factor (VEGF) receptor in lung cancer cells [56], and estrogen receptor α (ERα) in breast cancer cells [31]. Moreover, EGFR has been shown to regulate the expression of PD-L1 through the activation of the IL6/JAK/STAT3 pathway in NSCLC [57]. 17β-Estradiol-induced PD-L1 expression through ERα-mediated AKT signaling [58]. We suggest that not only is STAT3 activated through α9-nAChR, but the interaction of α9-nAChR with other receptors also regulates PD-L1 expression.

PD-L1 expression can be inducible or constitutive and may change over time in response to different stimuli such as EGF, cytokines, interferon-gamma (IFNγ), 17β-estradiol, BaP, DbA, and BzP [44,58,59]. In our study, we found that nicotine remarkably increased PD-L1 protein expression in a dose- and time-dependent manner through α9-nAChR (Figure 5), suggesting that nicotine-induced α9-nAChR activity can regulate anti-cancer immunity. In addition, nicotine-induced α9-nAChR stimulated cancer cell proliferation and migration to drive cancer development and progression (Figure 5). Our study suggested that a new role for nicotine in suppressing anticancer immunity through α9-nAChR-induced PD-L1 expression in melanoma.

The ERK, AKT and STAT3 signaling pathways have been previously described to regulate melanoma cell proliferation [2,60]. In this study, we found MEK, AKT and STAT3 inhibitors interfered with melanoma cell proliferation and attenuated nicotine-induced melanoma cell proliferation (Figure 3, Figure 4 and Figure 6). α9-nAChR regulated melanoma cell proliferation (Figure 3 and Figure 4). Stimulation of α9-nAChR with nicotine or overexpression of α9-nAChR promoted the activation of the AKT, ERK and STAT3 signaling pathways (Figure 3, Figure 5 and Figure 6). In contrast, knocking down α9-nAChR expression inhibited the AKT, ERK and STAT3 signaling pathways (Figure 4 and Figure 6). These observations, along with the rest of the results in this study, suggest an important role for the α9-nAChR-mediated AKT, ERK and STAT3 signaling pathways in regulating melanoma cell proliferation.

In a previous study, Chernyavsky et al. found that stimulation of α9-nAChR initiated cell migration in epidermal keratinocytes through activation of the phosphorylation of intercellular junction proteins (desmoglein 3 and β-catenin), and focal adhesion proteins (paxillin and focal adhesion kinase (FAK)), thereby separating cell-cell adhesion complexes; in contrast, knocking down α9-nAChR expression inhibited the phosphorylation of adhesion and cytoskeletal proteins [61]. Moreover, α9-nAChR regulated the shape and adhesion of keratinocytes through the activation of several kinases, including EGFR, Scr, PLC, and PKC, and GTPases (Rho and Rac) [61]. Recently, Chun et al. found that α9-nAChR in breast cancer cells interacted with EGFR and EEBB2, and these genes were coexpressed with numerous downregulated genes, such as nectin-3 (NECTIN3) in the adherens junction pathway [23]. In our present study, we also investigated the participation of α9-nAChR in the regulation of melanoma cell migration (Figure 3 and Figure 4), and stimulation of α9-nAChR by nicotine significantly increased melanoma cell migration (Figure 5). Furthermore, we found that the protein levels of the EMT markers Twist-1 and Snail-1 and morphological changes were affected by the overexpression or suppression of α9-nAChR (Figure 3 and Figure 4). These observations suggest that the α9-nAChR network is significantly associated with migration in melanoma cells.

Snail and Twist-1 transcription factors contribute to repression of the epithelial phenotype and activation of the mesenchymal phenotype [62]. Snail-1 and Twist-1 EMT markers are not enough for proving EMT program, but their expression is activated early in EMT, and they thus have central roles in metastatic cancer [62]. In many previous studied, researchers mentioned that AKT and EKR signaling pathways promote EMT markers, including Snail-1 and Twist-1 [62] and cigarette smoking-induced AKT and ERK signaling pathways promotes the EMT program in cancers [63]. Nicotine/ α9-nAChR activates AKT, ERK pathways and EMT markers in melanoma cells (Figure 3, Figure 4 and Figure 6). We suggest that nicotine/ α9-nAChR may induce the EMT program through AKT or ERK pathways, or both.

In this study, the expression of α9-nAChR was significantly associated with metastasis and OS. α9-nAChR had roles in inducing PD-L1 expression and regulating melanoma cell proliferation and migration. Notably, nicotine-induced α9-nAChR activity promoted melanoma cell proliferation through stimulation of the α9-nAChR-mediated AKT, ERK and STAT3 signaling pathways. In addition, nicotine-induced α9-nAchR activity promoted melanoma cell migration by activating the EMT pathway. PD-L1 expression was upregulated in melanoma cells after nicotine treatment via α9-nAchR-mediated activation of the binding of the transcription factor STAT3 to the PD-L1 promoter.

## 4. Materials and Methods

### 4.1. Cell Culture

The human primary neonatal foreskin melanocyte cell line HEMn-LP from a lightly pigmented donor was cultured at 37 °C and 5% CO2 in Medium 254 supplemented with human melanocyte growth supplement (HMGS). Melanocyte cell line, medium, and supplement listed above were purchased from Invitrogen (Carlsbad, CA, USA). The human melanoma cell line MDA-MB 435 was purchased from the American Tissue Cell Culture Collection (ATCC, Manassas, VA, USA), and the melanoma cell lines A2058 and A375 were obtained from Dr. Shing-Chuan Shen (Taipei Medical University, Taipei, Taiwan). All melanoma cell lines were maintained at 37 °C and 5% CO2 in Dulbecco’s modified Eagle’s medium (DMEM, Gibco, Grand Island, NY, USA) supplemented with 10% fetal bovine serum (FBS, Gibco) and 1% penicillin-streptomycin-neomycin (PSN) antibiotic mixture (Gibco).

### 4.2. Bioinformatics Analysis

#### 4.2.1. Melanoma Cell Line Dataset Analysis

Cell line datasets from the public R2 MegaSampler platform (https://hgserver1.amc.nl/cgi-bin/r2/main.cgi) were used to screen α9-nAChR mRNA expression in melanocyte, primary melanoma, and metastatic melanoma groups. We compared the α9-nAChR mRNA expression among the groups in the cell line datasets. Data from six different datasets in the melanoma cell phenotype-specific gene expression database (HOPP Database) (http://www.jurmo.ch/php/ genehunter.html) were used to assess α9-nAChR mRNA expression and PD-L1 expression in melanoma cell lines [45]. The melanoma cell lines from these datasets were divided into proliferative and invasive phenotypes. We conducted a statistical comparison of α9-nAChR mRNA expression values between the two phenotype groups using the nonparametric Mann-Whitney U-test. In addition, α9-nAChR and PD-L1 expression was used to divide the melanoma cell lines into high and low expression groups based on the mean level of α9-nAChR or PD-L1 expression. The χ^2^ test was used to analyze the correlation between α9-nAChR (low or high) and PD-L1 (low or high) expression. Pearson and Spearman’s rank correlation analysis was used to evaluate the strength of the association between α9-nAChR and PD-L1 expression. A *p*-value <0.05 was considered statistically significant.

#### 4.2.2. TCGA-SKCM Cohort Analysis

We downloaded gene expression data from the cancer genomic browser of UCSC (https://xenabrowser.net/). We selected gene expression of α9-nAChR and PD-L1, and then clinicopathological parameters (sex, age, ulceration, Breslow depth, Clerk level, tumor size, lymph node status, distant metastasis status, stage) were selected from the phenotype search option. All patient data were downloaded. Furthermore, OS time and events were also obtained. α9-nAChR and PD-L1 mRNA expression were used to divide the patients into high and low expression groups according to the mean level of α9-nAChR or PD-L1 mRNA expression. The χ^2^ test was used to analyze the correlations between α9-nAChR (low or high) mRNA expression, PD-L1 (low or high) mRNA expression, and clinicopathological parameters. Pearson and Spearman’s rank correlation analysis was used to evaluate the strength of the association between α9-nAChR and PD-L1 mRNA expression. A *p*-value <0.05 was considered statistically significant.

### 4.3. Kaplan-Meier Analysis

Kaplan-Meier analysis based on the result from publicly available R2: Kaplan Meier Scanner software (https://hgserver1.amc.nl), selected the TCGA-SKCM cohort, then selected the optimal cut-off value to separate α9-nAChR mRNA expression into high or low group, and finally selected OS. A *p*-value <0.05 was considered statistically significant.

### 4.4. Nicotine Treatment

Cells were seeded in 60 mm dishes or 6-well plates at 5 × 10^5^ cells per dish or 5 × 10^4^ cells per well, respectively. Nicotine was purchased from Sigma-Aldrich (St. Louis, MO, USA, Catalog #54-11-5). The final concentrations were 0.001, 0.001, 0.01, 0.1 and 1 μm/mL, and cells were treated for the indicated time periods. The cells were harvested and analyzed by a cell count assay and western blotting at the indicated time points.

### 4.5. Inhibitor Treatment

Cells were seeded at 5 × 10^5^ cells per dish in 60 mm dishes or 5 × 10^3^ cells per well in 96-well plates. These cells were pretreated with 10–25 μm LY 294002 (#1130), 10–25 μm PD 98059 (#1231), 2.5–5 μm Stattic (#2798) or 5–10 μm NSC74859 (#4655) for 3–24 h and then cultured in the presence or absence of nicotine (1 μm) for 24–48 h. All inhibitors were purchased from TOCRIS Bioscience (Minneapolis, MN, USA). The cells were harvested to assess cell proliferation and viability as well as protein expression, which was analyzed by western blotting.

### 4.6. Cell Proliferation and Viability Assays

#### 4.6.1. Cell Counting Assay

Cell numbers were determined by a cell counting assay. Cells were seeded in a 6-well plate at a density of 5 × 10^4^ cells per well and then incubated in medium for the indicated times. The cells were collected and suspended in a growth medium. After staining with trypan blue, the cell number was counted with a hemocytometer. This assay was repeated four times with duplicate samples.

#### 4.6.2. MTT Assay

Cell growth and viability were determined using a 3-(4,5-dimethylthiazol-2-yl)-2,5-diphenyltetrazolium (MTT) assay. Cells were seeded in triplicate in 96-well plates at a density of 5000 cells per well. Cells were treated with a compound at the indicated concentration for the indicated time periods. The medium was changed every two to three days. The optical density at 570 nm was measured using the SynergyTM HT Multi-Detection Microplate Reader (BioTek Instruments, Winooski, VT, USA). The assay was repeated three times with duplicate samples.

#### 4.6.3. Soft-Agar Growth Assay

Anchorage-independent cell growth was analyzed using a soft-agar colony formation assay. Cells were seeded in a 0.4% top agarose II (Amresco, Solon, OH, USA) solution in growth medium over a base layer of 0.9% agarose II in 6-well plates at a density of 1 × 10^4^ cells/mL. Soft-agar cultures were maintained at 37 °C for an additional 21 days and observed for the appearance of the colonies using the Leica DMI 4000 B Microscope Imaging System (Leica Microsystems, Wetzlar, Germany). The assay was repeated four times with duplicate samples. Images were acquired using a BioSpectrumTM 500 Imaging system, and colonies were counted under a light microscope.

### 4.7. Wound healing Scratch Assay

Cell migration was evaluated by a scratch-wound healing assay. In total, 5 × 10^5^ cells per well were seeded in 12-well plates. After the cells grew to approximately 90% confluence, the cell monolayers were wounded by manually scraping the monolayers with a 10 μL or 100 μL sterile pipette tips. Then cells were washed three times with 1× PBS and fresh medium was added. Migrated cells were observed and captured by using the Leica DMI 4000 B Microscope Imaging System. This assay was performed at 0 h and 24 h after wounding. Wound closure was quantified as a percentage (versus the control set to 100%) that indicated the relative distance between the two scratch edges (scratch wound) using ImageJ software.

### 4.8. Construction of Vectors

#### 4.8.1. α9-nAChR siRNA

The sequences of the α9-nAChR-scramble and α9-nAChR-siRNA primers are shown in Table S2. α9-nAChR-siRNA was constructed using two independent siRNAs. The scrambled siRNA sequence was used as a control. After BLAST analysis to verify the absence of significant sequence homology with other genes, the selected sequences were inserted into the *p*SUPER vector and digested with bglII and HindIII to generate the *p*SUPER-α9-nAChR-siRNA and *p*SUPER- α9-nAChR-scramble vectors.

#### 4.8.2. α9-nAChR Overexpression

The sequences of the α9-specific overexpression primers are shown in Table S2. After BLAST analysis to verify the absence of significant sequence homology with other human genes, the selected sequences were inserted into the pcDNA3.1 vector (Invitrogen) and digested with bglII and HindIII to generate pcDNA3.1-α9-nAChR overexpression vectors. The identity of the construct was confirmed by DNA sequence analysis.

### 4.9. Generation of Stable Cell Lines

For stable transfections, 5 × 10^6^ cells were washed twice with 1× PBS and mixed with 10 μg of vectors (*p*SUPER-α9-nAChR-siRNA, *p*SUPER-α9-nAChR-scramble, pcDNA3.1 or pcDNA3.1-α9 nAChR overexpression vector). The mixtures were transfected using the pipette-type microporator MP-100 electroporation (Invitrogen). After 48 h, the selection medium supplemented with G418 concentration of 5 mg/mL (A2058 cells) or 2 mg/mL (MDA-MB 435 cells) was added to the transfected cells. Every 2–3 days, the selection medium was replaced with new selection medium. After 30 days, the clones were isolated and evaluated for knockdown or overexpression at protein levels, and these clones were compared with scramble clones by western blotting.

### 4.10. RNA Isolation and RT-PCR Analysis

Total RNA was isolated from human cell lines using TRIzol (Invitrogen) according to the manufacturer’s protocol. The specific primers were synthesized as shown in Table S2. The PCR amplicons were analyzed on 1.2% agarose gels (Amresco) stained with ethidium bromide. Images were acquired by an INFINITY-a digital imaging system (Vilber Lourmet, France), and the intensities of the bands were quantified using ImageJ software. The relative mRNA levels were normalized against the GAPDH mRNA levels.

### 4.11. Protein Extraction, Western Blotting, and Antibody

To determine protein expression, we harvested cellular proteins for western blotting as previously described [20]. Cells were washed three times with ice-cold 1× PBS and lysed on ice in a cell lysis buffer (50 mM Tris-HCl, pH 8.0; 120 mM NaCl2; 0.5% NP-40; 100 mM sodium fluoride; and 200 M sodium orthovanadate) supplemented with protease inhibitors. Proteins (50 μg per lane) were separated by gel electrophoresis on 10% SDS-PAGE gels, transferred to nitrocellulose membrane by electroblotting. The membranes were probed with appropriate primary antibodies against α9-nAChR (Thermo Fisher Scientific, Rockford, IL, USA, #PA5-46826), PD-L1 (Genetex, Irvine, CA, USA, #GTX104763; Thermo Fisher Scientific, #14-5983-82; Santa Cruz Biotechnology, Santa Cruz, CA, USA, #sc-293425), total-AKT (Genetex, #GTX121937), phospho-AKT (Cell Signaling Technology, Beverly, MA, USA, #9271), total-ERK1/2 (Genetex, #GTX113094), phospho-ERK1/2 (Santa Cruz Biotechnology, #sc-7383), total-STAT3 (Santa Cruz Biotechnology, #sc-8059), phospho-STAT3 (Cell Signaling Technology, #9145), Snail-1 (Cell signaling Technology, #6615), Twist-1 (Santa Cruz Biotechnology, #sc-81417) and GAPDH (Genetex, #GTX627408) followed by incubation with an HRP-conjugated secondary antibody. Band intensity was quantified using ImageJ software. The relative protein levels were normalized against the GAPDH protein levels.

### 4.12. ChIP Assay

ChIP assays of the cultured cells were performed as described previously [31]. In brief, cells were cross-linked with a final concentration of 1% formaldehyde at 25 °C for 15 min and quenched with 0.125 M glycine for 5 min. Chromatin was sheared by sonication to average lengths of 500 bp. Chromatin was immunoprecipitated using 10 μg of anti-STAT3 monoclonal antibody (mAb) and protein A agarose beads at 4 °C overnight. DNA was recovered from the protein A complex using an elution buffer (10 mmol/L Tris, 10 mmol/L EDTA, 1% SDS, 150 mmol/L NaCl, and 5 mmol/L DTT, pH 8.0) and purified using a Macherey-Nagel PCR Clean-up kit. The DNA fragments were subjected to RT-PCR and qPCR using PD-L1 promoter primer pairs as shown in Supplemental Table S2. Nonimmunoprecipitated lysates (input) and immunoprecipitates obtained with an anti-rabbit IgG (Santa Cruz Biotechnology, #sc-2007) served as positive and negative controls, respectively. Relative enrichments of PD-L1 promoter region were calculated after normalization to GAPDH. Each of the immunoprecipitations was replicated three times, and each sample was quantified at least in triplicate.

### 4.13. IHC Staining and Scoring System

#### 4.13.1. IHC Staining

Tissue microarrays used in the present study were purchased from US Biomax, Inc. (Rockville, MD, USA). The tissue microarrays have 192 specimens, including 112 specimens of malignant melanoma, 64 specimens of metastatic malignant melanoma, eight specimens of adjacent normal skin tissue, and eight specimens of skin normal tissue. Each core of the tissue microarrays represents a specimen. US Biomax, Inc. also provided patient information concerning age, gender, clinical stage, TNM, and pathological diagnosis. Standard immunohistochemical procedures were implemented as follows: Paraffin sections were baked at 60 °C for 1h, de-paraffinized in xylene, rehydrated through graded ethanol, quenched for endogenous peroxidase activity in 3% hydrogen peroxide for 10 min, and processed for antigen retrieval by high pressure cooking in citrate antigen retrieval solution (pH = 6.0) for about 30 min. Sections was blocked with normal serum at room temperature for 30 min. Then, the samples on the slides were incubated at 37 °C for 2 h with rabbit polyclonal antibodies against α9-nAchR (1:100, Thermo Fisher Scientific, #PA5-77511) and mouse monoclonal antibodies against PD-L1 (1:100, Santa Cruz Biotechnology, #sc-293425) in a moist chamber. The slides were subsequently incubated with goat anti-Mouse/Rabbit IgG VisUCyte HRP Polymer Antibody (VC002, R&D Systems, Minneapolis, MN, USA) for 1 h at room temperature. Immunostaining was performed using the Liquid DAB-Substrate Chromogen System (K3468, Dako, Glostrup, Denmark), which resulted in a brown-colored precipitate at the antigen site. Subsequently, sections were counterstained with hematoxylin and the procedure of dehydration was implemented. Finally, covers slips were applied in a non-aqueous mounting medium.

#### 4.13.2. Immunohistochemistry Score

The staining scores were evaluated independently by two investigators, who were blinded to the information on the microarrays. For IHC analysis, the results were analyzed using Image-Pro Plus 6.0 software. The expression of each protein expression was evaluated by intensity scoring on scale of “0” to “3+” (0: no staining, +: weak staining, 2+: moderate staining, 3+: high staining) and proportion (0%, 1–24%, 25–50%, >50%). The final immunoreactive score was determined by multiplying the intensity score by the proportion of positive cells.

#### 4.14. Statistical Analysis

Data were analyzed using SPSS version 22.0 (SPSS Inc., Chicago, IL, USA) and SigmaPlot graphing software (San Jose, CA, USA). Continuous variables are presented as the mean ± standard deviation and were analyzed by two-tailed unpaired Student’s t-test for two groups. Nonmormally distributed data are expressed as the median and were assessed by the nonparametric Mann-Whitney U-test. The χ^2^ test analysis was used to study the associations between α9-nAChR (low or high) and PD-L1 (low or high) expression or different clinicopathological features. Pearson and Spearman’s rank correlation analysis was used for the association between α9-nAChR and PD-L1 expression levels. Kaplan-Meier analysis was used to plot survival curves, which were compared by the log-rank test. A *p*-value <0.05 (two-sided) was considered statistically significant.

## 5. Conclusions

**Figure 7:**
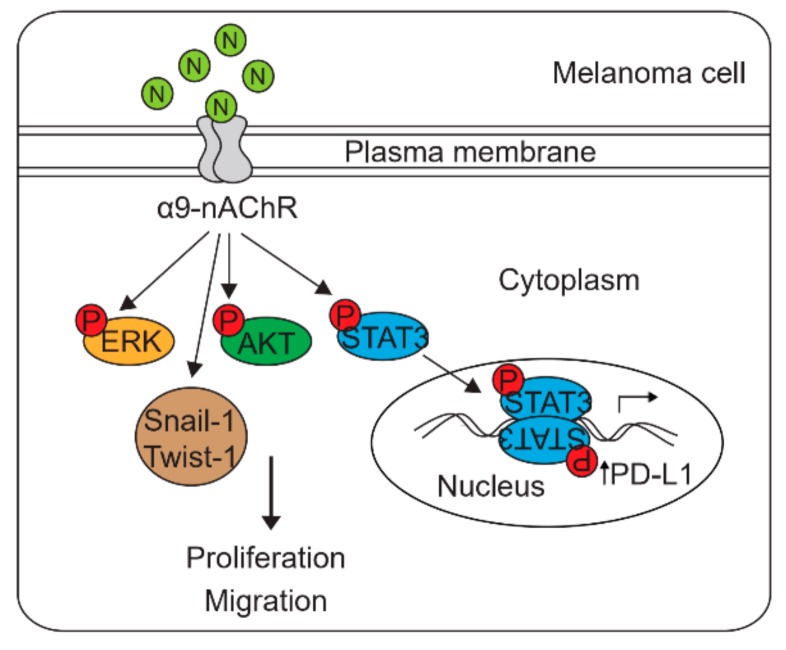
A schematic molecular model of nicotine-induced α9-nAChR activity in melanoma cells. α9-nAChR mediates nicotine-induced PD-L1 expression and regulates melanoma cell proliferation and migration. Nicotine-induced α9-nAChR activity upregulates Snail-1 and Twist-1 expression and activates the phosphorylation of AKT, ERK, and STAT3.

This study may reveal important insights into the mechanisms underlying nicotine-induced melanoma cell proliferation and migration mediated through α9-nAChR-initiated carcinogenic signaling and PD-L1 expression. α9-nAChR could serve as a therapeutic target to decrease the growth and metastasis in melanoma (Figure 7).

## Figures and Tables

**Table 1:** Correlations between α9-nAChR mRNA expression and clinicopathological parameters of melanoma patients determined using the TCGA-SKCM cohort (n = 472).

Parameters		CHRNA9 Low mRNA n (%)	CHRNA9 High mRNA n (%)	*p*-Value
Age (year)	≥40	147 (32%)	257 (55%)	0.33
	<40	18 (4%)	42 (9%)	
Sex	Female	114 (24%)	179 (38%)	0.04 *
	Male	53 (11%)	126 (27%)	
Ulceration	Yes	61 (19%)	106 (34%)	0.08
	No	40 (13%)	107 (34%)	
Breslow depth (mm)	≥2	76 (21%)	145 (40%)	0.8
	<2	46 (13%)	93 (26%)	
Clark level	I-III	32 (10%)	69 (21%)	0.24
	IV-V	85 (26%)	136 (42%)	
Tumor size	T1	14 (3%)	27 (7%)	0.87
	T2	27 (7%)	52 (13%)	
	T3	32 (8%)	59 (14%)	
	T4	60 (15%)	93 (23%)	
	Unknown	15 (4%)	32 (8%)	
Lymph node status	N0	94 (21%)	141 (31%)	0.035 *
	N1	20 (4%)	54 (12%)	
	N2	17 (4%)	32 (7%)	
	N3	22 (5%)	34 (8%)	
	Unknown	6 (1%)	30 (7%)	
Distant metastasis status	M0	148 (33%)	270 (61%)	0.39
	M1	10 (2%)	15 (3%)	
Stage	I-II	94 (25%)	123 (30%)	0.009 **
	III-IV	64 (15%)	131 (32%)	

Clinicopathological parameters were assessed using the χ^2^ test analysis. * *p* < 0.05, ** *p* < 0.01. We divided the patients into two groups according to the mean level of α9-nAChR mRNA expression. The first group expressed low levels of α9-nAChR, and the second group expressed high levels of α9-nAChR.

## Supplementary Materials

The following material is available online at https://www.mdpi.com/2072-6694/11/12/1991/s1, Table S1: The chi-square test was employed to assess the correlation between a9-nChAR and PD-L1 expression in the tissue microarrays (n = 192) as categorical variables, Table S2: RT-PCR primers, Figure S1: Uncropped scans with size marker indications of western blots shown in Figure 1D, Figure S2: α9-nAChR mRNA level in female (*n* = 179) and male (*n* = 293) groups, Figure S3: Correlation between α9-nAChR and PD-L1 mRNA expression in the melanoma cell line datasets, Figure S4: Correlation between α9-nAChR and PD-L1 mRNA expression in the TCGA-SKCM cohort, Figure S5: Correlations between α9-nAChR and PD-L1 in melanoma cell lines, Figure S6: Nicotine-induced α9-nAChR activity did not influence PD-L1 protein levels in the HEMn-LP melanocytes, and densitometry readings/intensity ratios, Figure S7: Uncropped scans with size marker indications of western blots shown in Figure 2L, and densitometry readings/intensity ratios, Figure S8: Uncropped scans with size marker indications of western blots shown in Figure 3A, and densitometry readings/intensity ratios, Figure S9: Uncropped scans with size marker indications of western blots shown in Figure 3J,L, and densitometry readings/intensity ratios, Figure S10: Uncropped scans with size marker indications of western blots shown in Figure 4A, and densitometry readings/intensity ratios, Figure S11: Uncropped scans with size marker indications of western blots shown in Figure 4G, and densitometry readings/intensity ratios, Figure S12: Uncropped scans with size marker indications of western blots shown in Figure 5B, and densitometry readings/intensity ratios, Figure S13: Uncropped scans with size marker indications of western blots shown in Figure 6A, and densitometry readings/intensity ratios, Figure S14: Uncropped scans with size marker indications of western blots shown in Figure 6B, and densitometry readings/intensity ratios, Figure S15: Uncropped scans with size marker indications of western blots shown in Figure 6D,F, and densitometry readings/intensity ratios, Figure S16: Uncropped scans with size marker indications of western blots shown in Figure 6H,J, and densitometry readings/intensity ratios, Figure S17: Uncropped scans with size marker indications of western blots shown in Figure 6K, and densitometry readings/intensity ratios, Figure S18: Uncropped scans with size marker indications of western blots shown in Figure S6, and densitometry readings/intensity ratios.
