# Supplementary material for: The α9 Nicotinic Acetylcholine Receptor Mediates Nicotine-Induced PD-L1 Expression and Regulates Melanoma Cell Proliferation and Migration
Source: Cancers (Basel). 2019 Dec 11;11(12):1991. doi: 10.3390/cancers11121991 (PMC6966517; doi:10.3390/cancers11121991)
Supplement: Supplementary file 1 [file cancers-11-01991-s001.pdf]

Supplementary Materials: The α9 Nicotinic Acetylcholine Receptor Mediates Nicotine-Induced PD-L1 Expression and Regulates Melanoma Cell Proliferation and Migration

Hai Duong Nguyen, You-Cheng Liao, Yuan-Soon Ho, Li-Ching Chen, Tzu-Chun Cheng, Hui-Wen Chang, Donald Liu, Woan-Ruoh Lee, Shing-Chuan Shen, Chih-Hsiung Wu and Shih-Hsin Tu

Table S1. The chi-square test was employed to assess the correlation between a9-nChAR and PD-L1 expression in the tissue microarrays (*n* = 192) as categorical variables.

Tissue microarrays	α9-nAChR low mRNA *n* (%)	α9-nAChR high mRNA *n* (%)	*p*-value
PD-L1 low mRNA - n (%)	79 (81%)	25 (26%)	1.8×10^{-14}
PD-L1 high mRNA - n (%)	18 (19%)	70 (74%)	

Table S2. RT-PCR primers.

Primer name	Primer sequence
α1-nAChR	F: 5′-GAGTCTAACAATGCGGCG-3′<br>R: 5′-TTTCTGCTCATCCTTGCTG-3′
α2-nAChR	F: 5′-TCCTGTGTTCCTGTCCTTCA-3′<br>R: 5′-GGTCCTCAGTCTCGGTATG-3′
α3-nAChR	F: 5′-GAGAAGGTGACCCTGTG-3′<br>R: 5′-GTGATGACGATGGACAAGGTTA-3′
α4-nAChR	F: 5′-GTTGGCGTATTTGCGTC-3′<br>R: 5′-GAGAGGTCAATCCACGG-3′
α5-nAChR	F: 5′-ACGCTTCCCAAACTGCT-3′<br>R: 5′-CTTCAACAACCTCACGG-3′
α6-nAChR	F: 5′-ACGCTTTGTATTTCAGTCCT-3′<br>R: 5′-GGGTGCGGTAGTGTATG-3′
α7-nAChR	F: 5′-CGGCAAGAGGAGTGAAA-3′<br>R: 5′-GAACACCAGCAGGGCGA-3′
α9-nAChR	F: 5′-AGGGTGGTCATCCTGAAAT-3′<br>R: 5′-AGGTCTTTGTTCCTGGC-3′
α10-nAChR	F: 5′-CACAAGGGAGCACTCAT-3′<br>R: 5′-CCAATACCCAGCACAAAC-3′
PD-L1	F: 5′-AGG TGC GTT CAG ATG TTG GC-3′<br>R: 5′-TGC CCA AGG CAG CAA ATC CAG-3′
GAPDH	F: 5′-ATCCCATCACCATCTTCCAG-3′<br>R: 5′-GCTGATGATCTTGAGGCTGT-3′
PD-L1 promotor	F: 5′-GAC CTG GCT GCA CTA ATT GTC-3′<br>R: 5′-CAT TTC CCA GGG AGA GCT GG-3′
α9-nAChR-siRNA-1	F: 5′-GATCCCCCACCAATGTGGTCCTGCGGTTCAAGAGA CCGCAGGACCACATTGGTGTTTTTA-3′<br>R: 5′-AGCTTAAAAACACCAATGTGGTCCTGCGGTCTCTT GAACCGCAGGACCACATTGGTGGGG-3′
α9-nAChR-siRNA-2	F: 5′-GATCCCCAGACCACAAGGCCACCAATTTCAAG AGAATTGGTGGCCTTGTGGTCTTTTTA-3′<br>R: 5′-AGCTTAAAAAAGACCACAAGGCCACCAATT CTCTTGAAATTGGTGGCCTTGTGGTCTGGG-3′

α9-nAChR-scramble	F: 5′-GATCCCCGAGAACACGAATACACCCCTTCAA GAGAGGGGTGTATTCGTGTTCTCTTTTTA-3′<br>R: 5′-AGCTTAAAAAGAGAACACGAATACACCCCTCTC TTGAAGGGGTGTATTCGTGTTCTCGGG-3′
pcDNA3.1 to α9-nAChR overexpression	F: 5′-AATAAGCTTGCCGCCACCATGAACTGGTCCCATTCCTG-3′<br>R: 5′-ATAGAATTCACATCCGCTCTTGCTATGA-3′

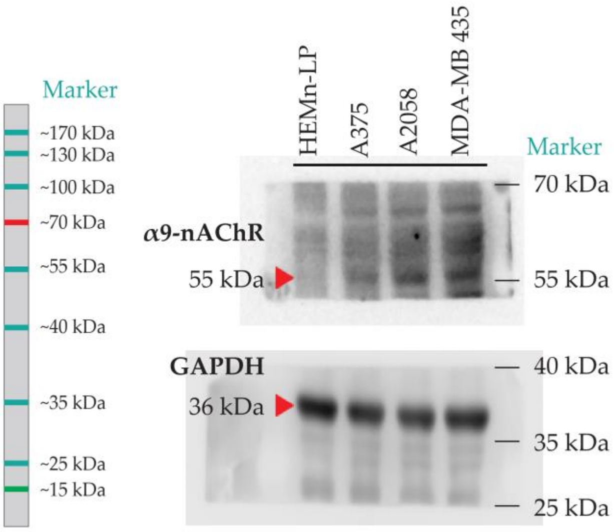

Densitometric values

	HEMn -LP	A375	A2058	MDA-MB 435
GAPDH	21,929	19,693	18,808	23,696
α9-nAChR	6,450	13,363	17,431	26,977
Ratio	0.29	0.68	0.93	1.14

Figure S1. Uncropped scans with size marker indications of western blots shown in Figure 1D, and densitometry readings/intensity ratios.

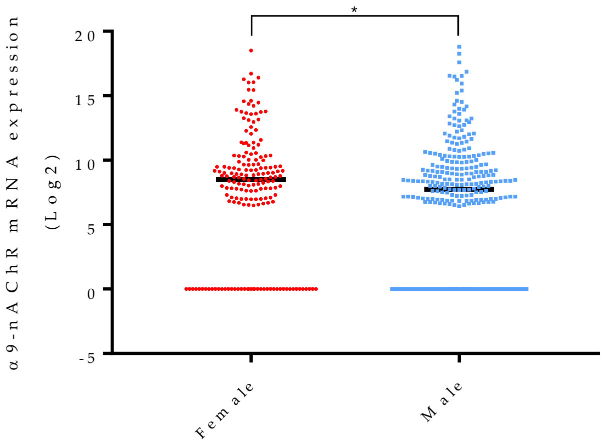

Figure S2. α9-nAChR mRNA level in female (n = 179) and male (n = 293) groups. The result was analyzed from TCGA-SKCM cohort (n = 472). The data were analyzed by the Mann-Whitney test. The median of α9-nAChR expression in each group is shown by the horizontal line; * $p < 0.05$.

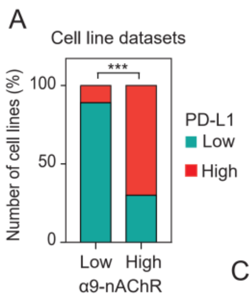

B

Melanoma cell line datasets	α9-nAChR low mRNA - *n* (%)	α9-nAChR high mRNA - *n* (%)	*p*-value
PD-L1 low mRNA n (%)	139 (89%)	6 (30%)	6.5×10^{-9}
PD-L1 high mRNA n (%)	17 (11%)	14 (70%)	

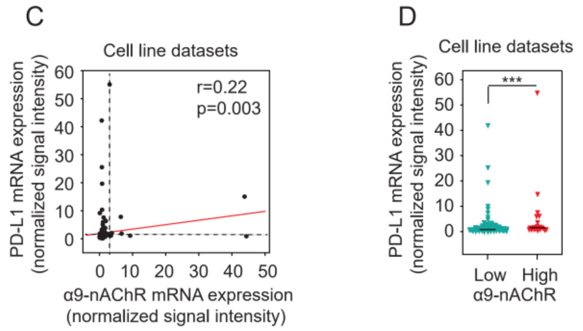

Figure S3. Correlation between α9-nAChR and PD-L1 mRNA expression in the melanoma cell line datasets. **(A, B)** The association between α9-nAChR and PD-L1 mRNA levels in the melanoma cell line datasets (n = 176) as categorical variables. The χ^2 test was employed to assess the correlation between a9-nAChR and PD-L1 expression in samples. ** $p < 0.01$. **(C)** Correlation between α9-nAChR and PD-L1 mRNA expression in the melanoma cell line datasets (n = 176). Pearson and Spearman's rank correlation measured the strength of the association between α9-nAChR and PD-L1 expression. **(D)** PDL1 mRNA expression in melanoma cell lines with low (n = 156) or high (n = 20) α9-nAChR expression in the melanoma cell line datasets. The data were analyzed by the Mann-Whitney test. The median α9-nAChR expression in each group is shown by a horizontal line. *** $p < 0.001$.

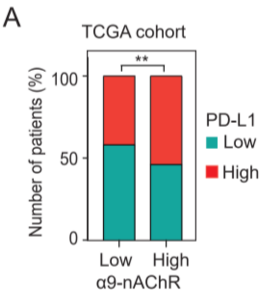

B

TCGA-SKCM cohort	α9-nAChR low mRNA - *n* (%)	α9-nAChR high mRNA - *n* (%)	*p*-value
PD-L1 low mRNA n (%)	97 (58%)	139 (46%)	0.009
PD-L1 high mRNA n (%)	70 (42%)	166 (54%)	

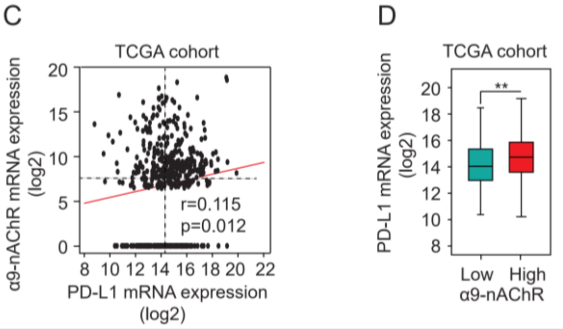

Figure S4. Correlation between α9-nAChR and PD-L1 mRNA expression in the TCGA-SKCM cohort. **(A, B)** The association between α9-nAChR and PD-L1 mRNA levels in the TCGA-SKCM cohort (n=472) as categorical variables. The χ^2 test was employed to assess the correlation between a9-nAChR and PD-L1 expression in samples. ** $p < 0.01$. **(C)** Correlation between α9-nAChR and PD-L1 mRNA expression in the TCGA-SKCM cohort (n = 472). Pearson and Spearman's rank correlation measured the strength of the association between α9-nAChR and PD-L1 expression. **(D)** PD-L1 mRNA expression in patients in the TCGA-SKCM cohort with high (n = 305) or low α9-nAChR (n = 167) expression. The data are presented as the mean ± SD, ** $p < 0.01$, Student's t-test.

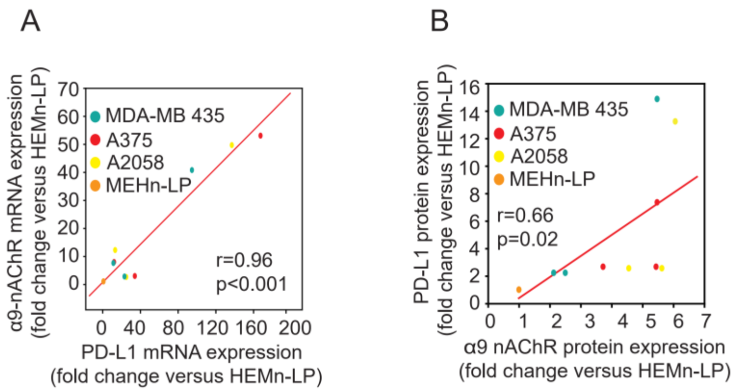

Figure S5. Correlations between α9-nAChR and PD-L1 in melanoma cell lines. **(A, B)** Correlations between α9-nAChR and PD-L1 mRNA and protein levels in the HEMn-LP, A375, A2058, and MDA-MB 435 cell lines. The experiments were repeated three times per cell line. Band intensities were measured using ImageJ software and the amounts of α9-nAChR and PD-L1 at the mRNA and protein levels in the A375, A2058, and MDA-MB 435 melanoma cells were normalized to the α9-nAChR and PD-L1 mRNA and protein levels in the HEMn-LP cells.

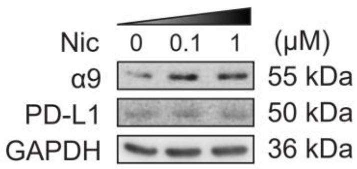

Figure S6. Nicotine-induced α9-nAChR activity did not influence PD-L1 protein levels in the HEMn-LP melanocytes. The results were determined by western blotting.

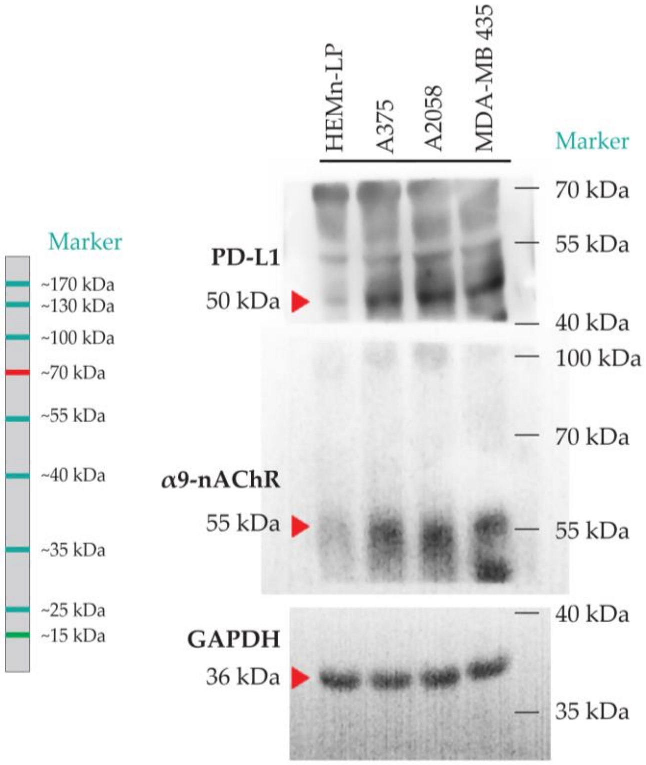

Densitometric values

	HEMn-LP	A375	A2058	MDA-MB 435
GAPDH	21,931	18,980	21,703	23,572
PD-L1	4,829	24,753	26,825	27,778
Ratio	0.22	1.30	1.24	1.18
α9-nAChR	9,027	23,536	22,767	24,239
Ratio	0.41	1.24	1.05	1.03

Figure S7. Uncropped scans with size marker indications of western blots shown in Figure 2L, and densitometry readings/intensity ratios.

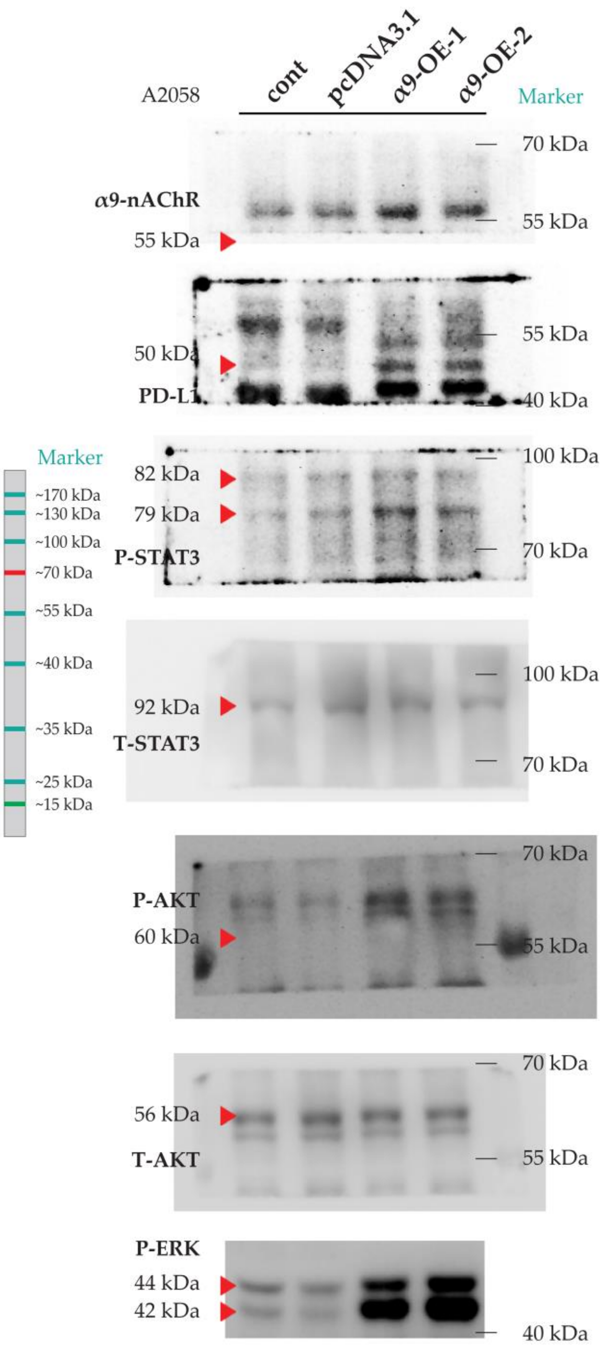

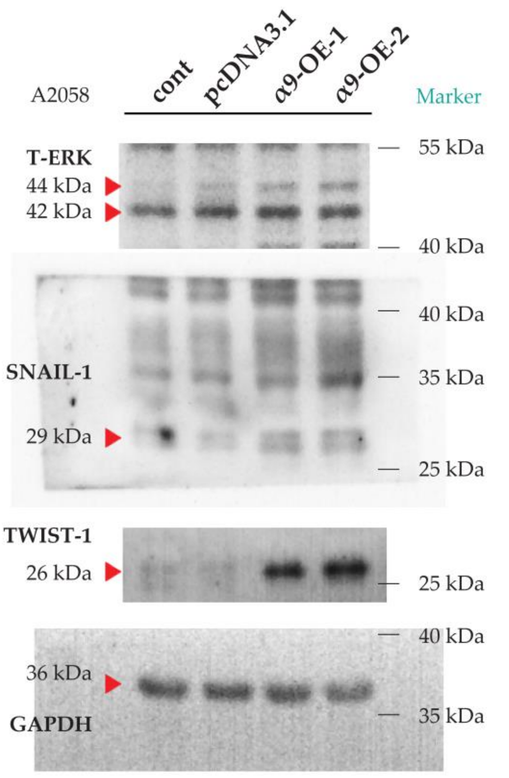

Densitometric values

A2058	cont	pcDNA3.1	α9-OE-1	α9-OE-2
GAPDH	21,493	18,367	16,181	14,458
α9-nAChR	15,415	17,511	24,260	22,876
Ratio	0.72	0.95	1.50	1.58
PD-L1	10,010	10,826	25,250	25,422
Ratio	0.47	0.59	1.56	1.76
P-STAT3	9,141	11,250	21,991	19,677
Ratio	0.43	0.61	1.36	1.36
T-STAT3	9,912	12,992	11,881	11,535
Ratio	0.46	0.71	0.73	0.80
P-AKT	9,761	7,968	24,452	25,941
Ratio	0.45	0.43	1.51	1.79
T-AKT	16,980	21,042	18,006	21,741
Ratio	0.79	1.15	1.11	1.50
P-ERK	5,075	4,520	28,099	31,567
Ratio	0.24	0.25	1.74	2.18
T-ERK	30,559	28,750	30,436	27,093
Ratio	1.42	1.57	1.88	1.87
SNAIL-1	6,947	7,133	15,157	17,432
Ratio	0.32	0.39	0.94	1.21
TWIST-1	5,741	5,422	19,804	26,743
Ratio	0.27	0.30	1.22	1.85

Figure S8. Uncropped scans with size marker indications of western blots shown in Figure 3A, and densitometry readings/intensity ratios.

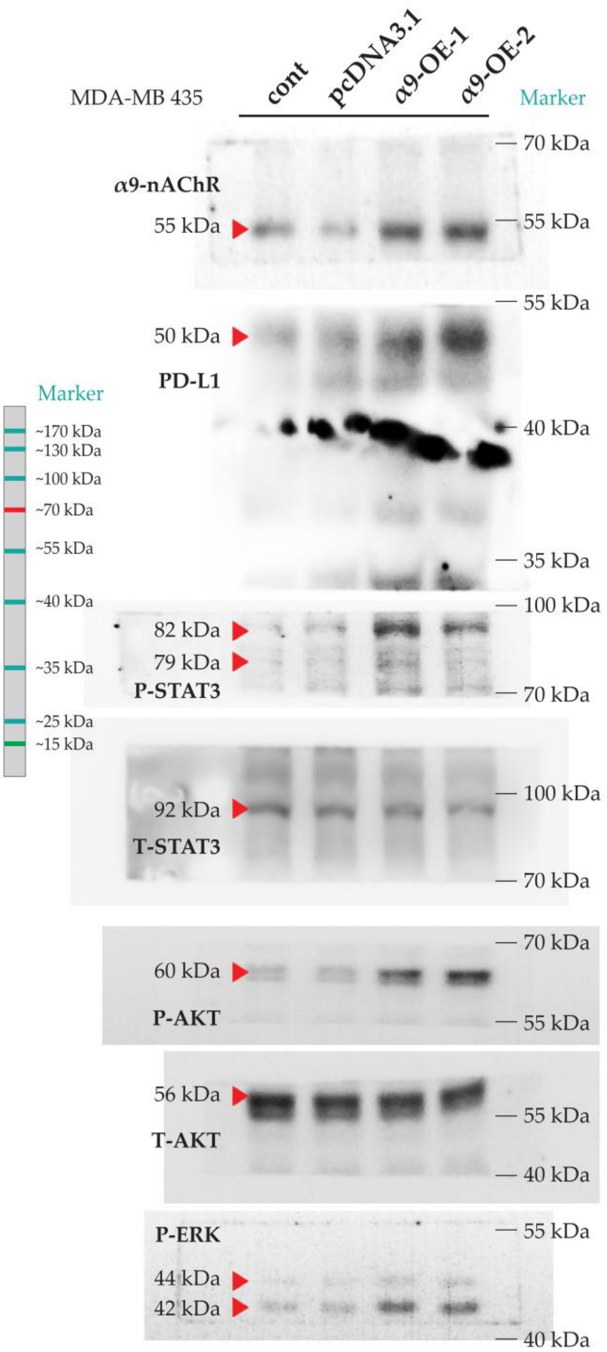

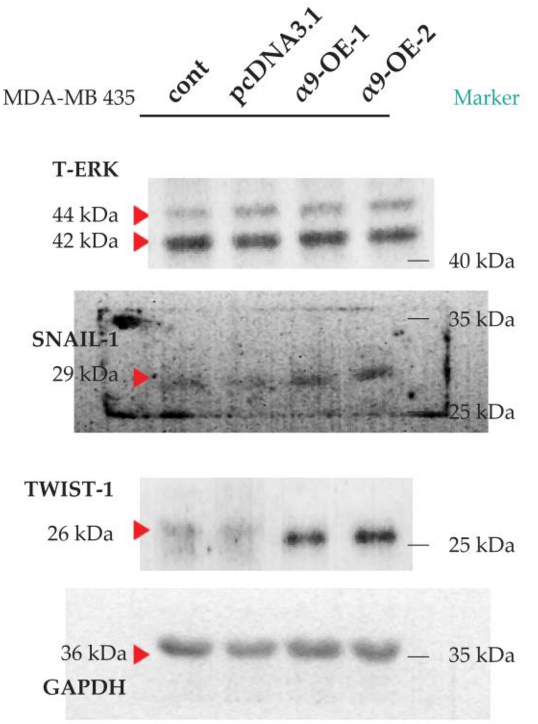

Densitometric values

MDA-MB 435	cont	pcDNA3.1	α9-OE-1	α9-OE-2
GAPDH	19,684	12,431	17,131	15,319
α9-nAChR	14,268	7,855	25,328	29,579
Ratio	0.72	0.63	1.48	1.93
PD-L1	8,510	7,902	20,762	27,552
Ratio	0.43	0.64	1.21	1.80
P-STAT3	3,545	7,353	25,844	15,763
Ratio	0.18	0.59	1.51	1.03
T-STAT3	23,688	18,161	19,558	16,716
Ratio	1.20	1.46	1.14	1.09
P-AKT	6,540	6,090	20,616	26,832
Ratio	0.33	0.49	1.20	1.75
T-AKT	26,727	23,206	21,127	20,096
Ratio	1.36	1.87	1.23	1.31
P-ERK	5,847	6,133	19,873	17,074
Ratio	0.30	0.49	1.16	1.11
T-ERK	34,603	27,767	31,743	29,664
Ratio	1.76	2.23	1.85	1.94
SNAIL-1	13,926	9,364	19,536	19,259
Ratio	0.71	0.75	1.14	1.26
TWIST-1	13,335	9,798	23,918	25,393
Ratio	0.68	0.79	1.40	1.66

Figure S8. Uncropped scans with size marker indications of western blots shown in Figure 3A (continued), and densitometry readings/intensity ratios.

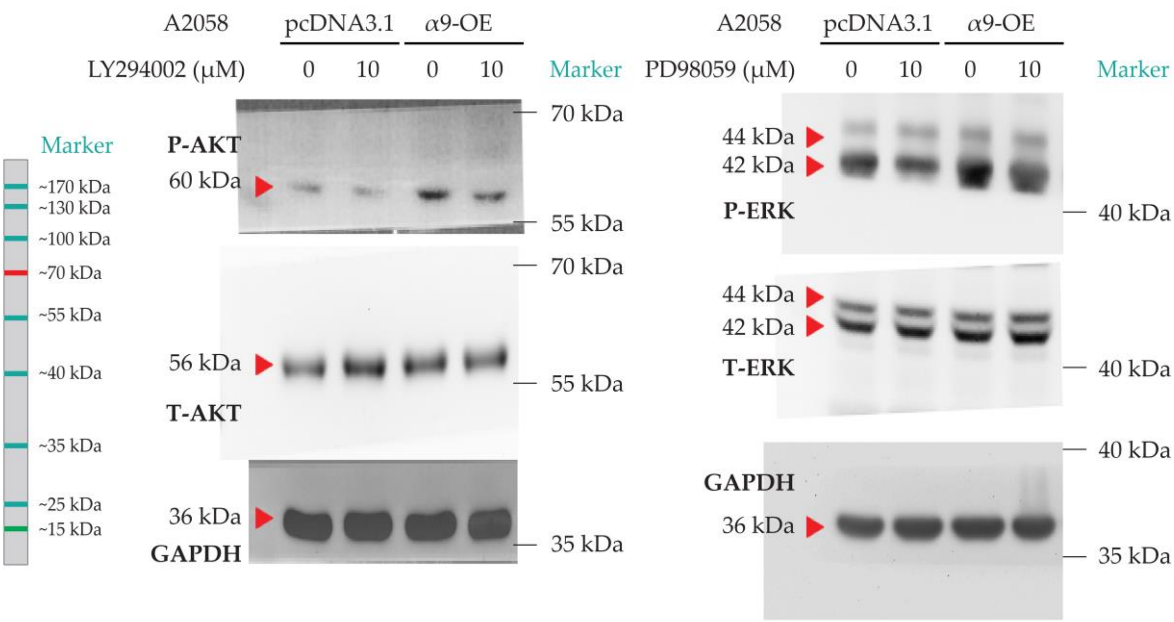

Densitometric values

A2058<br>LY294002 (μM)	pcDNA3.1<br>0	 10	α9-OE<br>0	 10
GAPDH	30,380	35,521	35,193	30,764
P-AKT	10,450	7,831	23,210	14,815
Ratio	0.34	0.22	0.66	0.48
T-AKT	27,051	29,212	25,913	23,596
Ratio	0.89	0.82	0.74	0.77

Densitometric values

A2058<br>PD98059 (μM)	pcDNA3.1<br>0	 10	α9-OE<br>0	 10
GAPDH	28,607	30,948	26,456	28,840
P-ERK	16,431	14,667	22,437	18,555
Ratio	0.57	0.47	0.85	0.64
T-ERK	24,073	25,446	27,090	29,594
Ratio	0.84	0.82	1.02	1.03

Figure S9. Uncropped scans with size marker indications of western blots shown in Figure 3J and Figure 3L, and densitometry readings/intensity ratios.

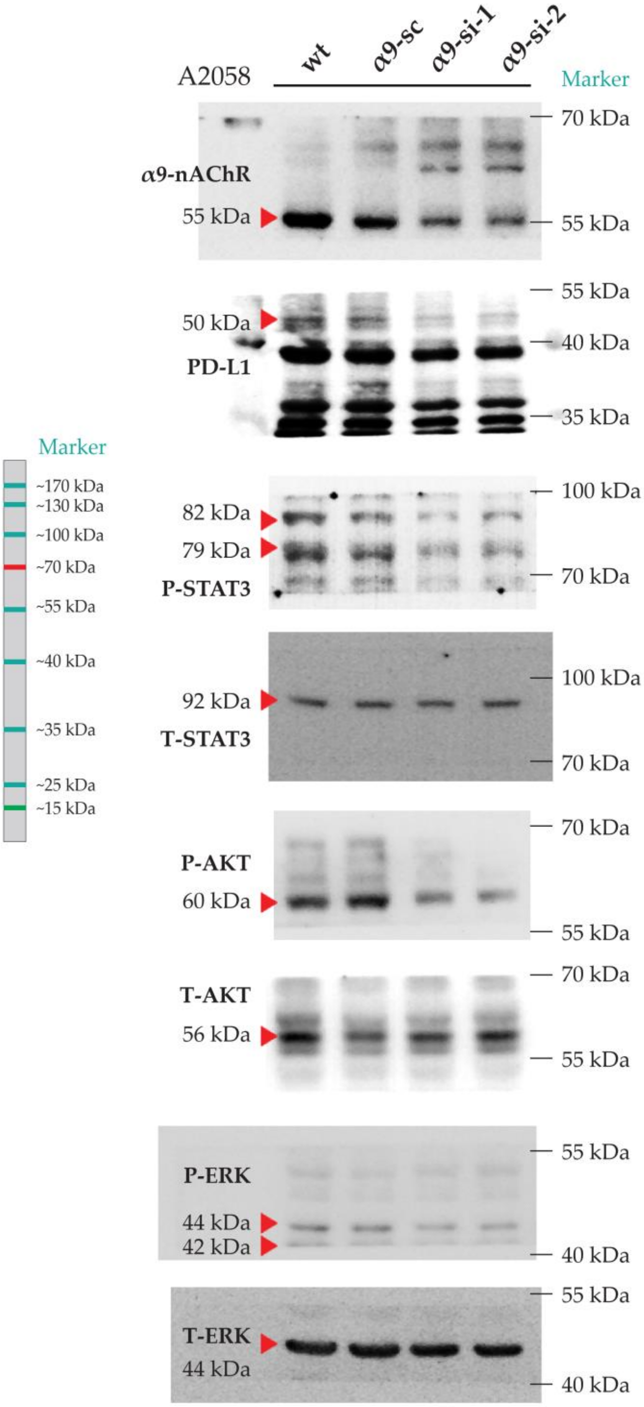

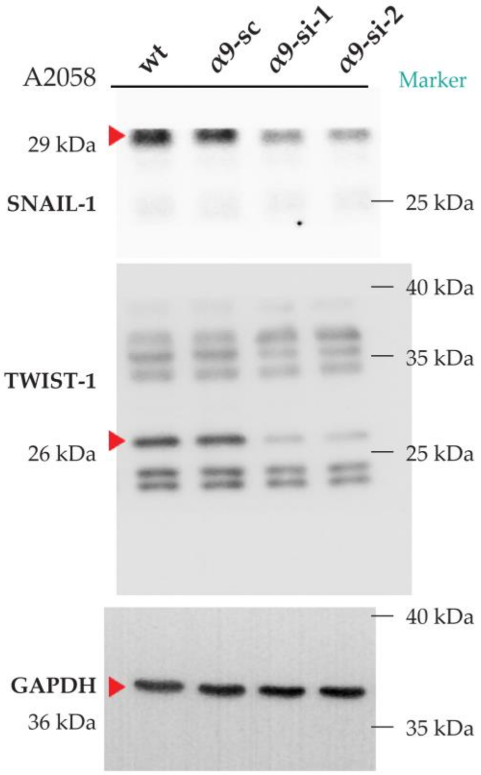

Densitometric values

A2058	wt	α9-sc	α9-si-1	α9-si-2
GAPDH	21,899	23,570	23,843	24,814
α9-nAChR	29,970	22,273	13,625	13,951
Ratio	1.37	0.94	0.57	0.56
PD-L1	29,884	20,650	7,616	6,113
Ratio	1.36	0.88	0.32	0.25
P-STAT3	25,313	20,592	8,972	10,972
Ratio	1.16	0.87	0.38	0.44
T-STAT3	22,330	22,709	20,030	20,623
Ratio	1.02	0.96	0.84	0.83
P-AKT	22,322	27,438	11,306	7,733
Ratio	1.02	1.16	0.47	0.31
T-AKT	29,538	20,413	21,838	24,664
Ratio	1.35	0.87	0.92	0.99
P-ERK	22,293	18,435	10,277	11,148
Ratio	1.02	0.78	0.43	0.45
T-ERK	30,528	29,479	27,328	27,020
Ratio	1.39	1.25	1.15	1.09
SNAIL-1	28,823	25,079	11,438	9,745
Ratio	1.32	1.06	0.48	0.39
TWIST-1	29,054	30,384	4,704	5,573
Ratio	1.33	1.29	0.20	0.22

Figure S10. Uncropped scans with size marker indications of western blots shown in Figure 4A, and densitometry readings/intensity ratios.

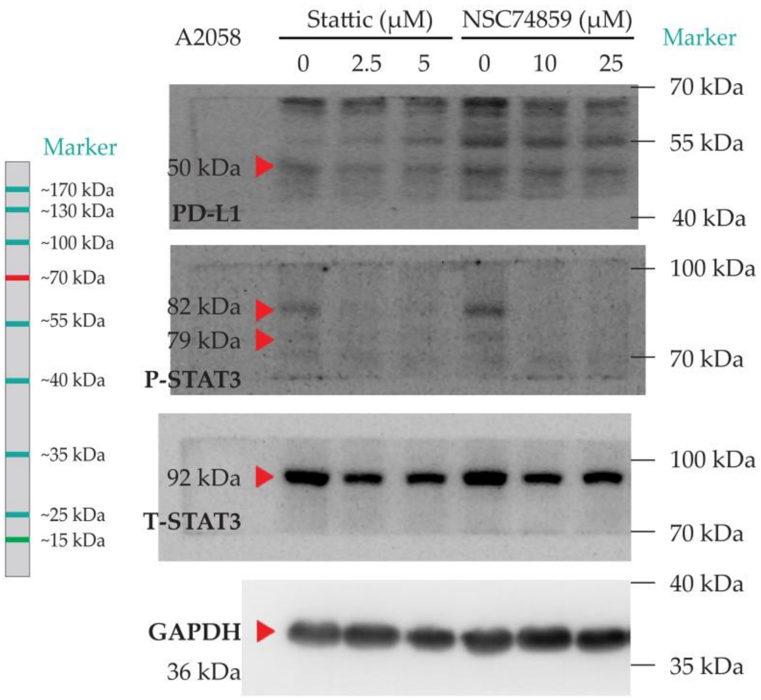

Densitometric values

A2058 Stattic	Concentration (μM) 0	2.5	5
GAPDH	7,432	9,392	9,898
PD-L1	13,879	9,417	9,602
Ratio	1.87	1.00	0.97
P-STAT3	11,720	6,385	6,791
Ratio	1.58	0.68	0.69
T-STAT3	18,855	11,468	12,224
Ratio	2.54	1.22	1.24

Densitometric values

A2058 NSC74859	Concentration (μM) 0	10	25
GAPDH	10,095	10,499	10,262
PD-L1	17,457	13,828	12,001
Ratio	1.73	1.32	1.17
P-STAT3	17,653	5,412	3,408
Ratio	1.75	0.52	0.33
T-STAT3	17,932	11,671	12,320
Ratio	1.78	1.11	1.20

Figure S11. Uncropped scans with size marker indications of western blots shown in Figure 4G, and densitometry readings/intensity ratios.

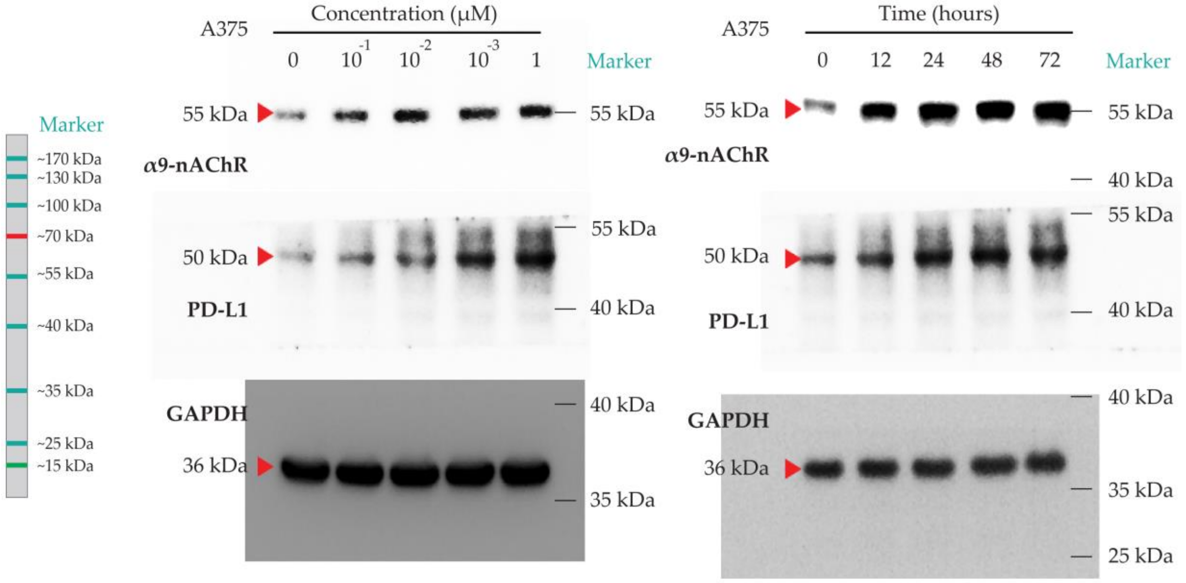

Densitometric values

A375	Nicotine (μM) 0	10^{-3}	10^{-2}	10^{-1}	1
GAPDH	15,875	16,884	17,607	16,973	16,665
α9-nAChR	6,995	14,331	19,486	18,995	18,453
Ratio	0.44	0.85	1.11	1.12	1.11
PD-L1	3,939	7,026	11,600	16,856	20,873
Ratio	0.25	0.42	0.66	0.99	1.25

Densitometric values

A375	Nicotine (1μM) 0	12	24	48	72
GAPDH	16,394	20,505	19,399	17,443	16,030
α9-nAChR	3,973	14,801	19,022	18,667	19,611
Ratio	0.24	0.72	0.98	1.07	1.22
PD-L1	9,123	15,569	21,502	22,852	22,325
Ratio	0.56	0.76	1.11	1.31	1.39

Figure S12. Uncropped scans with size marker indications of western blots shown in Figure 5B, and densitometry readings/intensity ratios.

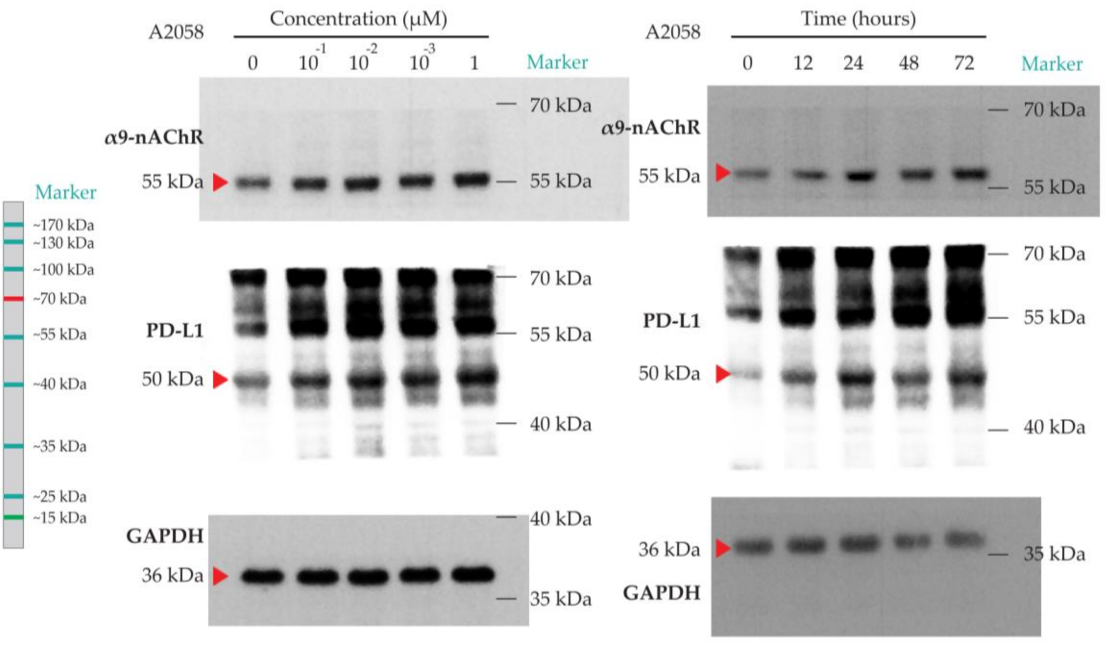

Densitometric values

A2058	Nicotine (μM)				
	0	10^{-3}	10^{-2}	10^{-1}	1
GAPDH	11,130	10,578	10,934	11,223	11,948
α9-nAChR	6,747	8,661	9,571	9,100	10,488
Ratio	0.61	0.82	0.88	0.81	0.88
PD-L1	4,671	8,775	11,466	10,941	12,954
Ratio	0.42	0.83	1.05	0.97	1.08

Densitometric values

A2058	Nicotine (1μM)				
	0	12	24	48	72
GAPDH	15,927	18,321	20,687	12,698	13,988
α9-nAChR	3,973	14,801	19,022	18,667	19,611
Ratio	0.25	0.81	0.92	1.47	1.40
PD-L1	1,916	5,538	8,415	7,070	9,687
Ratio	0.12	0.30	0.41	0.56	0.69

Figure S12. Uncropped scans with size marker indications of western blots shown in Figure 5B (continued), and densitometry readings/intensity ratios.

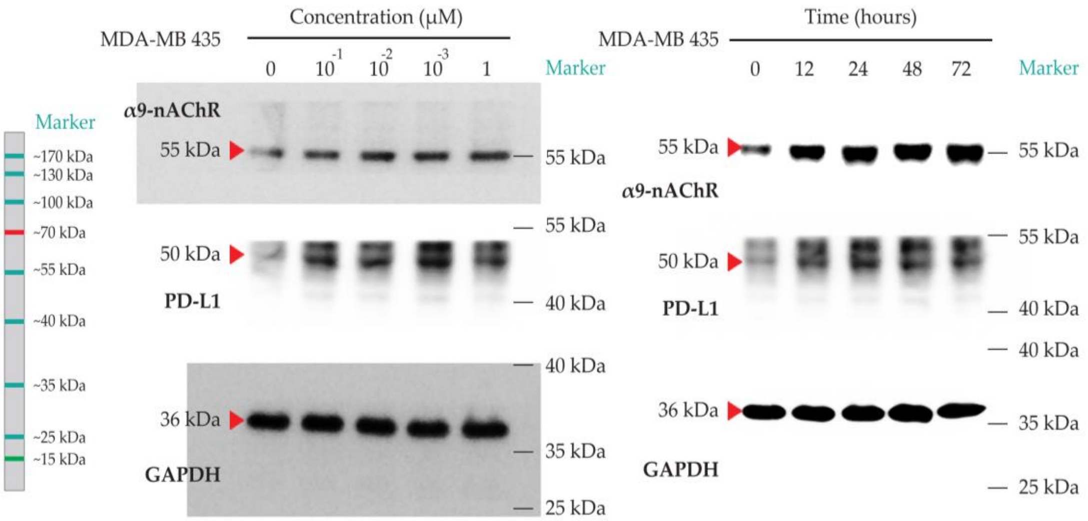

Densitometric values

MDA-MB 435	Nicotine (μM)				
	0	10^{-3}	10^{-2}	10^{-1}	1
GAPDH	9,994	10,703	10,116	10,166	11,973
α9-nAChR	4,914	7,085	8,626	8,234	9,768
Ratio	0.49	0.66	0.85	0.81	0.82
PD-L1	2,678	7,816	7,686	10,045	7,567
Ratio	0.27	0.73	0.76	0.99	0.63

Densitometric values

MDA-MB 435	Nicotine (1μM)				
	0	12	24	48	72
GAPDH	22,850	23,585	23,510	26,694	23,304
α9-nAChR	7,173	18,787	21,600	22,683	24,169
Ratio	0.31	0.80	0.92	0.85	1.04
PD-L1	4,652	8,912	9,691	9,814	8,383
Ratio	0.20	0.38	0.41	0.37	0.36

Figure S12. Uncropped scans with size marker indications of western blots shown in Figure 5B (continued), and densitometry readings/intensity ratios.

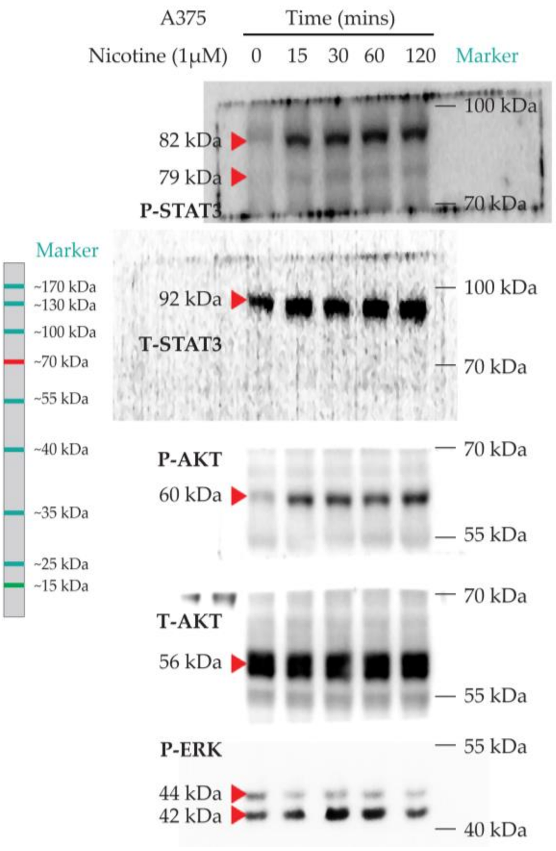

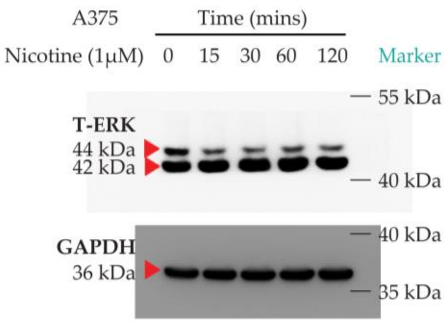

Densitometric values

A375	Nicotine (1μM)				
	**0**	**15**	**30**	**60**	**120**
GAPDH	15,661	16,713	17,636	17,020	16,855
P-STAT3	6,594	16,114	15,919	17,345	17,675
Ratio	0.42	0.96	0.90	1.02	1.05
T-STAT3	14,656	20,359	18,531	19,090	22,523
Ratio	0.94	1.22	1.05	1.12	1.34
P-AKT	8,997	19,663	19,727	18,920	23,851
Ratio	0.57	1.18	1.12	1.11	1.42
T-AKT	23,051	21,934	21,893	22,337	23,600
Ratio	1.47	1.31	1.24	1.31	1.40
P-ERK	11,856	11,903	18,440	16,041	9,835
Ratio	0.76	0.71	1.05	0.94	0.58
T-ERK	20,331	19,254	19,119	19,482	19,899
Ratio	1.30	1.15	1.08	1.14	1.18

Figure S13. Uncropped scans with size marker indications of western blots shown in Figure 6A, and densitometry readings/intensity ratios.

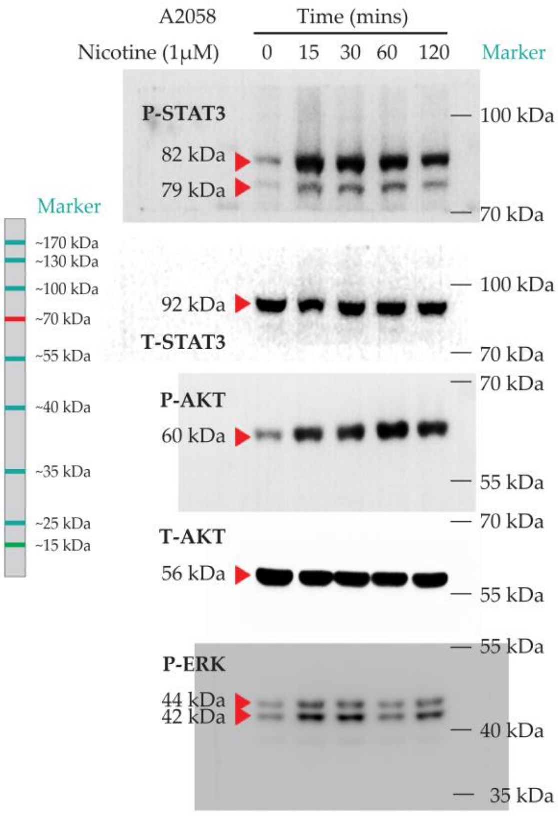

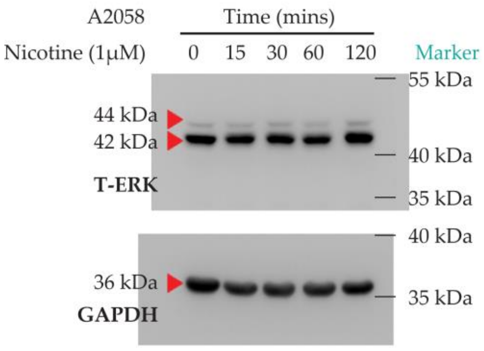

Densitometric values

A2058	Nicotine (1μM)				
	**0**	**15**	**30**	**60**	**120**
GAPDH	17,253	12,051	14,628	15,011	16,812
P-STAT3	3,046	16,671	14,700	14,669	10,815
Ratio	0.18	1.38	1.00	0.98	0.64
T-STAT3	18,361	17,829	18,981	18,568	15,865
Ratio	1.06	1.48	1.30	1.24	0.94
P-AKT	5,960	15,417	15,226	22,106	17,588
Ratio	0.35	1.28	1.04	1.47	1.05
T-AKT	17,674	16,719	20,873	15,330	19,107
Ratio	1.02	1.39	1.43	1.02	1.14
P-ERK	6,024	17,568	16,665	7,635	14,644
Ratio	0.35	1.46	1.14	0.51	0.87
T-ERK	15,076	14,317	14,422	13,562	16,658
Ratio	0.87	1.19	0.99	0.90	0.99

Figure S13. Uncropped scans with size marker indications of western blots shown in Figure 6A (continued), and densitometry readings/intensity ratios.

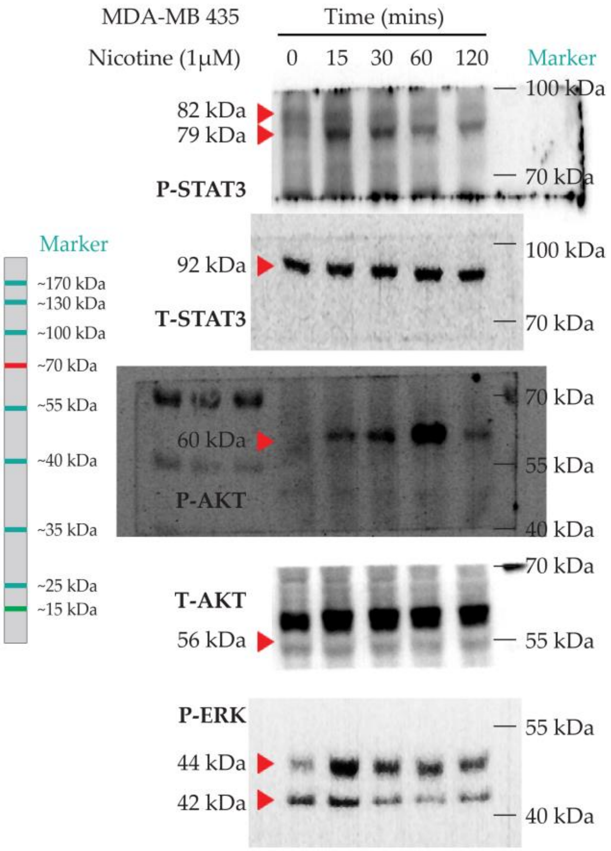

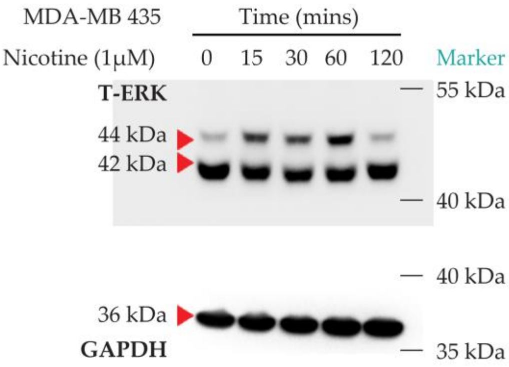

MDA-MB 435	Nicotine (1μM)				
	**0**	**15**	**30**	**60**	**120**
GAPDH	15,553	16,813	17,520	16,588	15,540
P-STAT3	14,422	21,593	17,471	13,079	11,542
Ratio	0.93	1.28	1.00	0.79	0.74
T-STAT3	14,108	12,790	13,313	12,468	12,381
Ratio	0.91	0.76	0.76	0.75	0.80
P-AKT	6,477	11,451	13,398	24,881	9,956
Ratio	0.42	0.68	0.76	1.50	0.64
T-AKT	19,462	23,925	21,895	21,251	22,621
Ratio	1.25	1.42	1.25	1.28	1.46
P-ERK	9,411	21,294	12,433	10,877	12,245
Ratio	0.61	1.27	0.71	0.66	0.79
T-ERK	15,550	17,342	15,786	19,418	15,115
Ratio	1.00	1.03	0.90	1.17	0.97

Figure S13. Uncropped scans with size marker indications of western blots shown in Figure 6A (continued), and densitometry readings/intensity ratios.

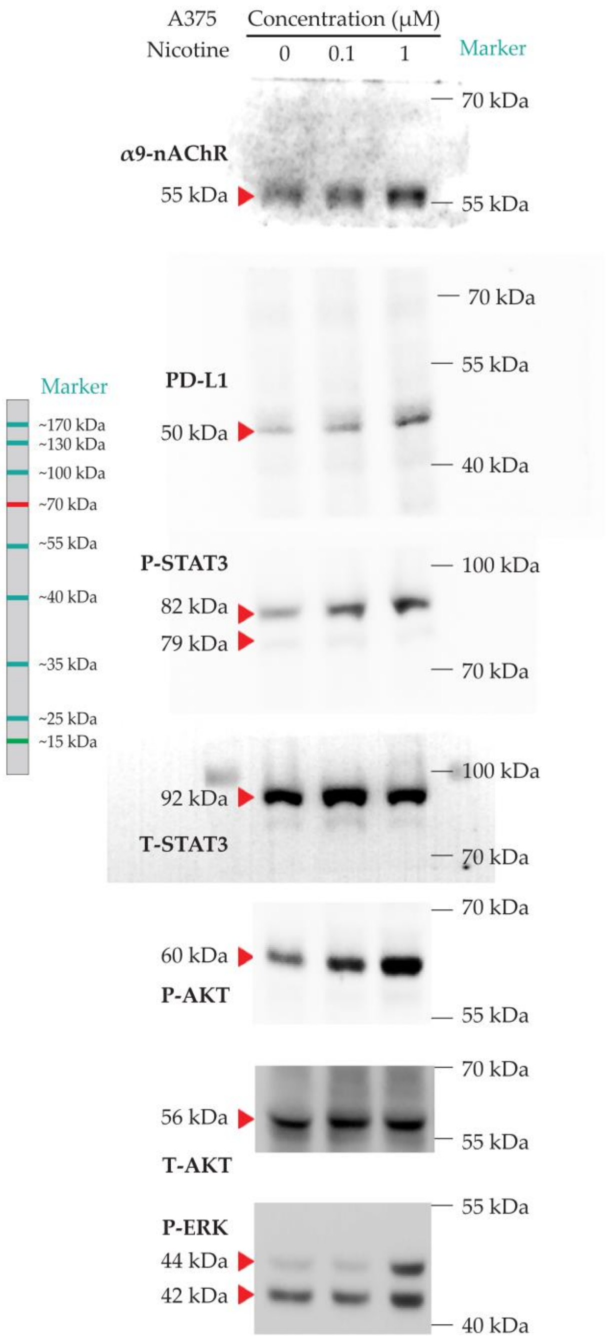

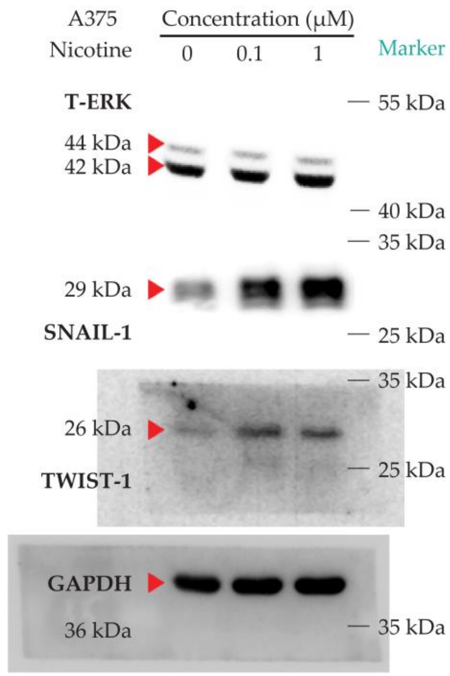

Densitometric values

**A375 Nicotine**	**Concentration (μM) 0**	**0.1**	**1**
GAPDH	26,743	30,716	31,863
α9-nAChR	14,149	18,744	25,221
Ratio	0.53	0.61	0.79
PD-L1	11,781	19,057	27,364
Ratio	0.44	0.62	0.86
P-STAT3	8,926	15,174	17,031
Ratio	0.33	0.49	0.53
T-STAT3	27,164	32,576	26,741
Ratio	1.02	1.06	0.84
P-AKT	15,060	28,596	44,398
Ratio	0.56	0.93	1.39
T-AKT	35,641	40,470	37,851
Ratio	1.33	1.32	1.19
P-ERK	16,941	21,685	35,767
Ratio	0.63	0.71	1.12
T-ERK	31,938	33,965	36,624
Ratio	1.19	1.11	1.15
SNAIL-1	12,918	33,058	39,630
Ratio	0.48	1.08	1.24
TWIST-1	5,920	22,282	20,636
Ratio	0.22	0.73	0.65

Figure S14. Uncropped scans with size marker indications of western blots shown in Figure 6B, and densitometry readings/intensity ratios.

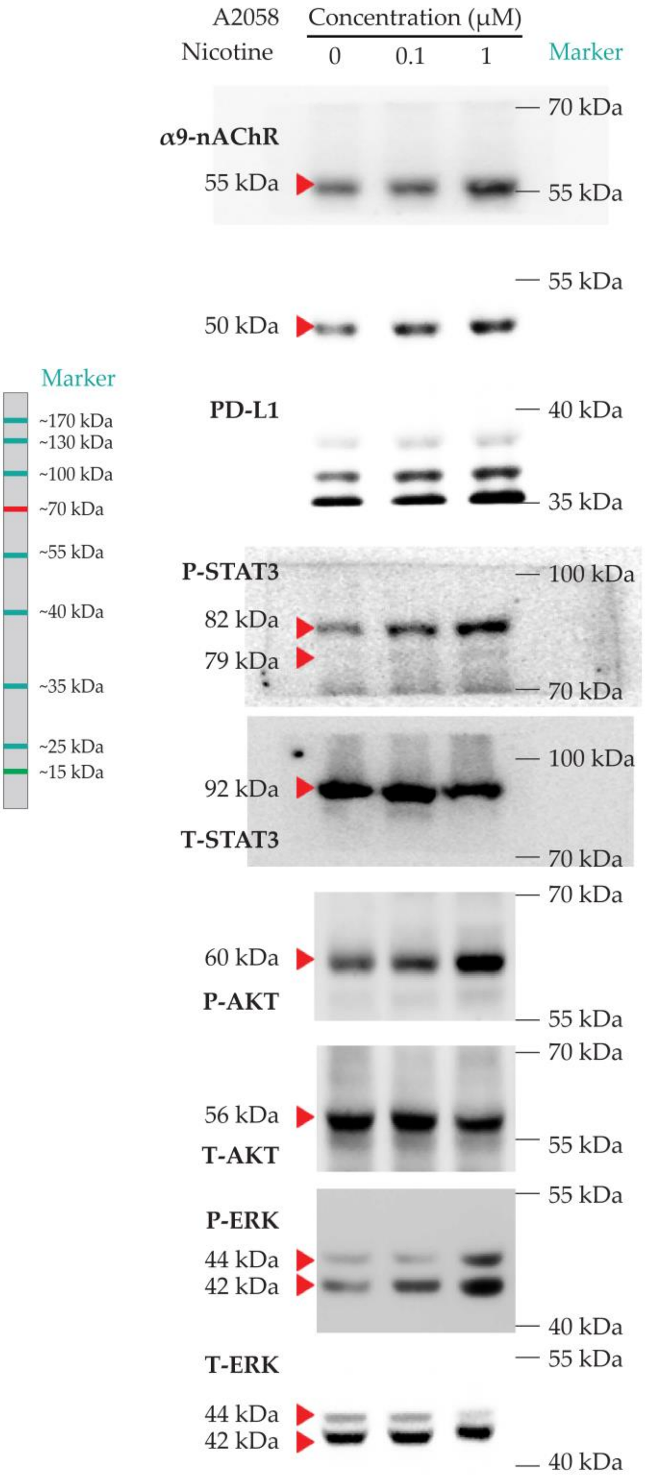

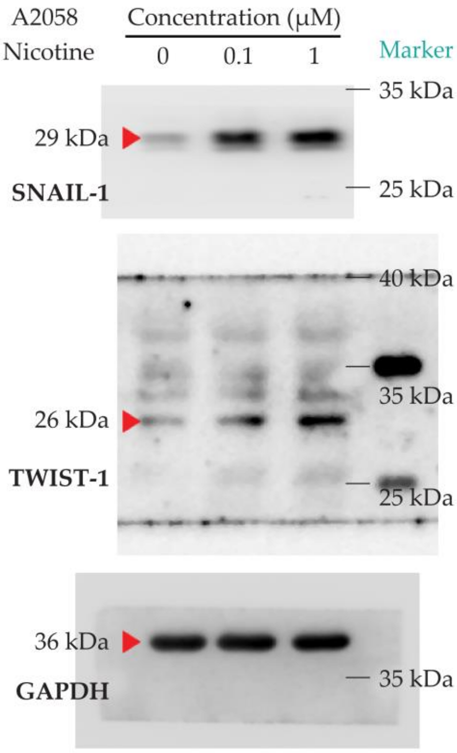

Densitometric values

A2058 Nicotine	Concentration (μM)		
	0	0.1	1
GAPDH	31,765	32,106	34,849
α9-nAChR	21,781	24,356	37,568
Ratio	0.69	0.76	1.08
PD-L1	16,637	25,810	30,713
Ratio	0.52	0.80	0.88
P-STAT3	16,121	26,655	37,172
Ratio	0.51	0.83	1.07
T-STAT3	29,780	27,948	23,632
Ratio	0.94	0.87	0.68
P-AKT	24,444	31,462	47,727
Ratio	0.77	0.98	1.37
T-AKT	42,210	43,362	37,724
Ratio	1.33	1.35	1.08
P-ERK	10,105	17,802	32,763
Ratio	0.32	0.55	0.94
T-ERK	40,725	43,202	34,762
Ratio	1.28	1.35	1.00
SNAIL-1	7,006	31,023	36,736
Ratio	0.22	0.97	1.05
TWIST-1	10,326	20,686	34,224
Ratio	0.33	0.64	0.98

Figure S14. Uncropped scans with size marker indications of western blots shown in Figure 6B (continued), and densitometry readings/intensity ratios.

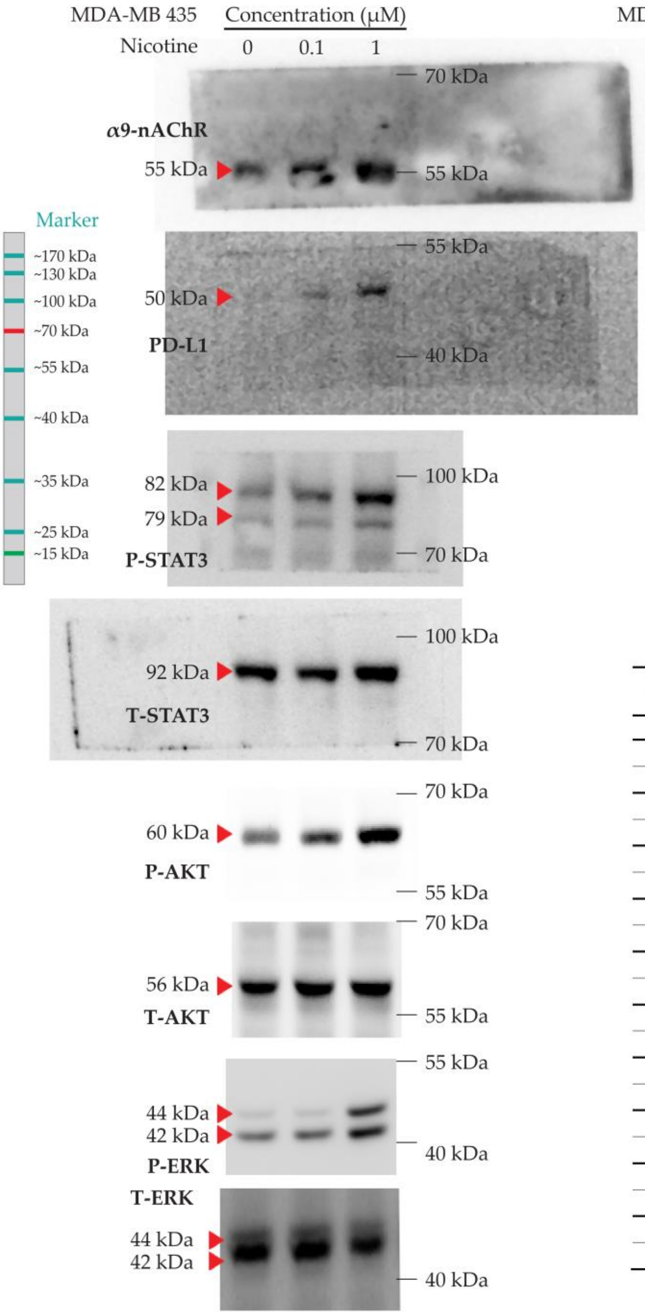

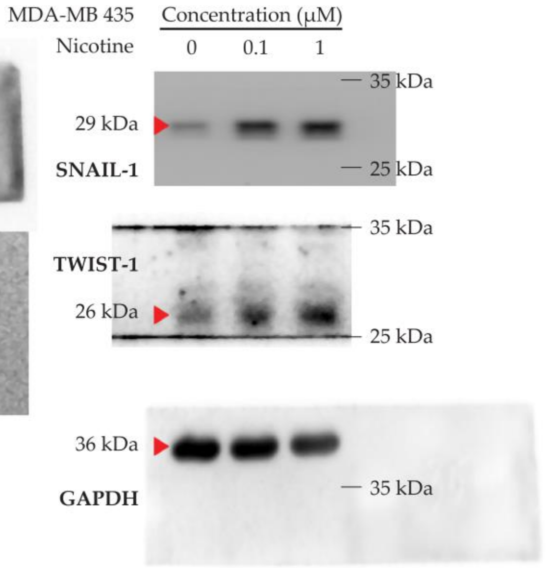

Densitometric values

**MDA-MB 435**	**Concentration (μM)**		
**Nicotine**	**0**	**0.1**	**1**
GAPDH	38,962	35,636	30,010
α9-nAChR	12,929	30,141	33,325
Ratio	0.33	0.85	1.11
PD-L1	3,945	12,546	23,805
Ratio	0.10	0.35	0.79
P-STAT3	15,261	22,321	36,584
Ratio	0.39	0.63	1.22
T-STAT3	26,976	23,302	28,286
Ratio	0.69	0.65	0.94
P-AKT	20,035	27,144	44,938
Ratio	0.51	0.76	1.50
T-AKT	45,880	44,910	45,159
Ratio	1.18	1.26	1.50
P-ERK	12,883	14,975	39,971
Ratio	0.33	0.42	1.33
T-ERK	38,139	37,243	29,780
Ratio	0.98	1.05	0.99
SNAIL-1	8,735	29,602	32,231
Ratio	0.22	0.83	1.07
TWIST-1	21,026	26,509	35,534
Ratio	0.54	0.74	1.18

Figure S14. Uncropped scans with size marker indications of western blots shown in Figure 6B (continued), and densitometry readings/intensity ratios.

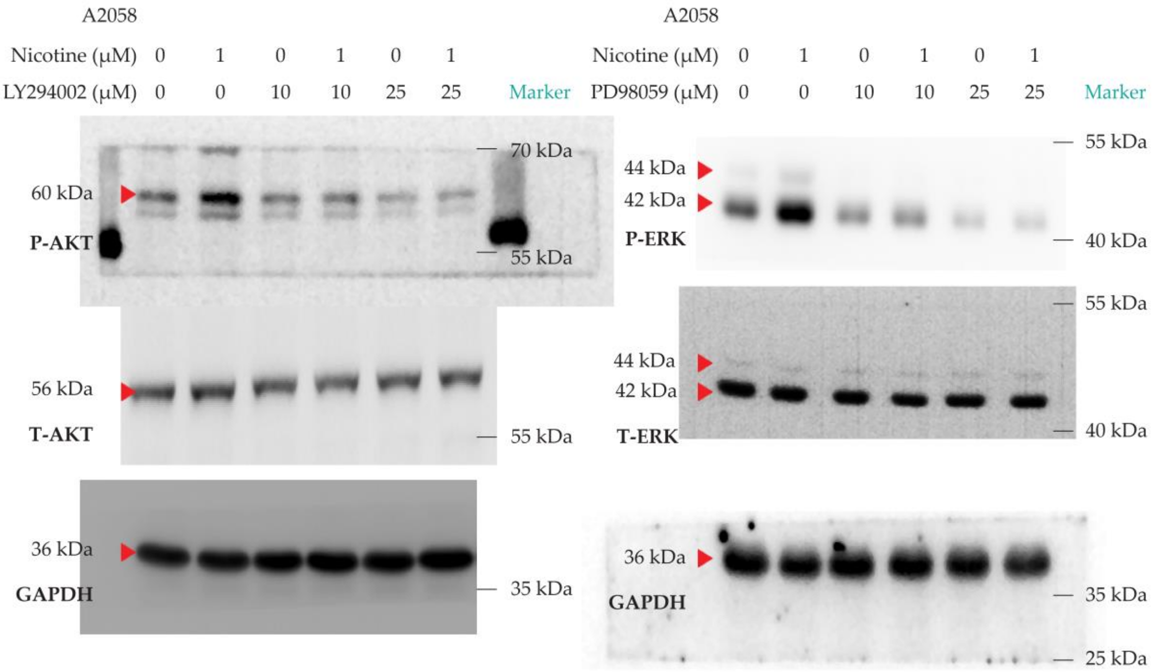

Densitometric values

A2058	Concentration (μM)					
**Nicotine**	**0**	**1**	**0**	**1**	**0**	**1**
**LY294002**	**0**	**0**	**10**	**10**	**25**	**25**
GAPDH	13,425	13,972	14,809	14,793	14,082	15,682
P-AKT	10,451	19,639	8,575	8,697	5,557	5,082
Ratio	0.78	1.41	0.58	0.59	0.39	0.32
T-AKT	17,014	18,563	17,548	15,700	17,270	16,726
Ratio	1.27	1.33	1.18	1.06	1.23	1.07

Densitometric values

A2058	Concentration (μM)					
**Nicotine**	**0**	**1**	**0**	**1**	**0**	**1**
**PD98059**	**0**	**0**	**10**	**10**	**25**	**25**
GAPDH	11,680	10,001	14,019	13,937	13,076	13,863
P-ERK	8,169	16,458	4,510	5,010	1,832	1,660
Ratio	0.70	1.65	0.32	0.36	0.14	0.12
T-ERK	13,429	12,731	14,355	12,574	15,303	14,470
Ratio	1.15	1.27	1.02	0.90	1.17	1.04

Figure S15. Uncropped scans with size marker indications of western blots shown in Figure 6D and Figure 6F, and densitometry readings/intensity ratios.

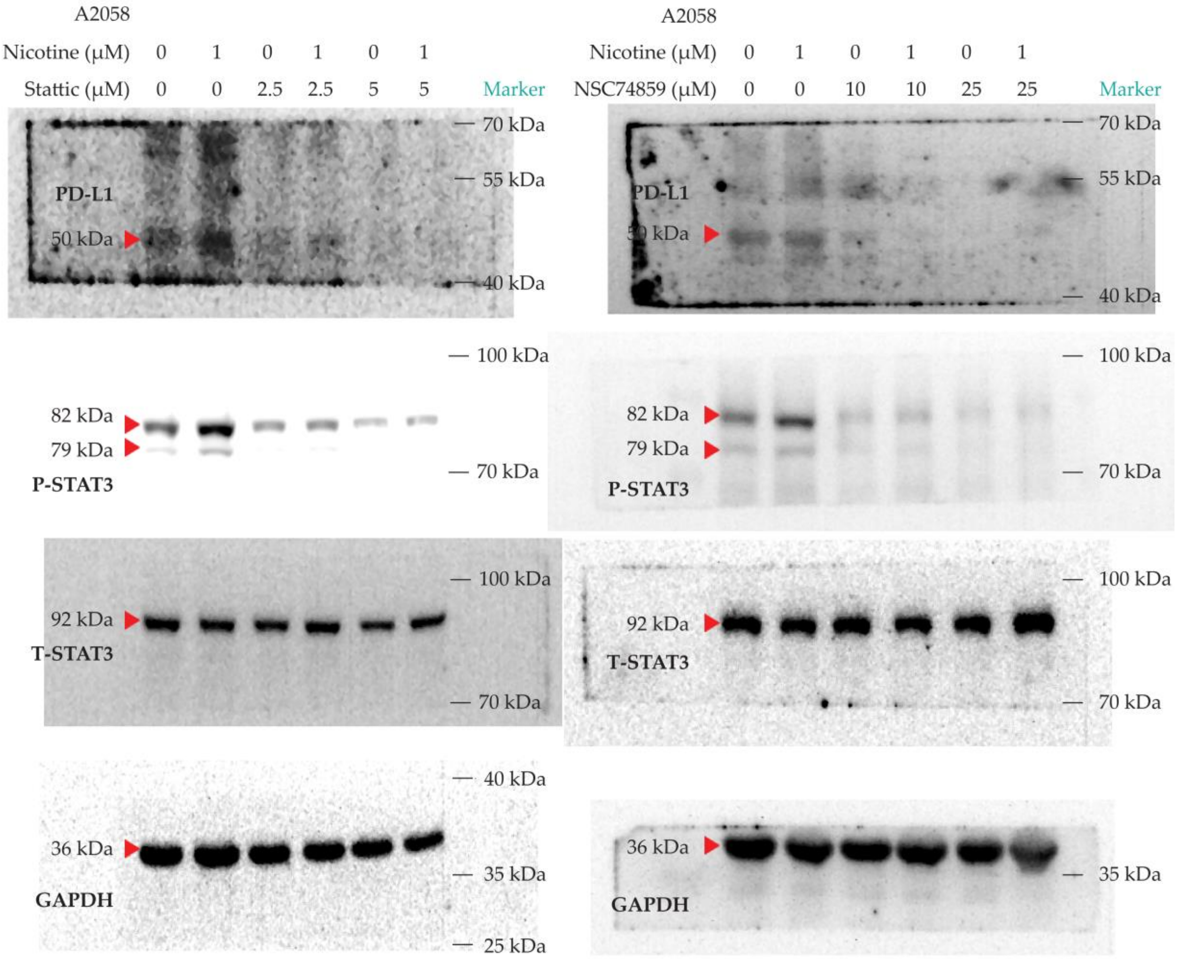

Densitometric values

A2058	Concentration (μM)					
**Nicotine**	**0**	**1**	**0**	**1**	**0**	**1**
**Stattic**	**0**	**0**	**2.5**	**2.5**	**5**	**5**
GAPDH	16,048	18,214	14,849	13,496	12,179	11,865
PD-L1	8,478	12,938	6,096	5,870	3,609	3,549
Ratio	0.53	0.71	0.41	0.43	0.30	0.30
P-STAT3	7,192	15,112	3,020	3,445	1,367	1,580
Ratio	0.45	0.83	0.20	0.26	0.11	0.13
T-STAT3	17,411	15,542	13,338	15,851	10,926	14,213
Ratio	1.08	0.85	0.90	1.17	0.90	1.20

Densitometric values

A2058	Concentration (μM)					
**Nicotine**	**0**	**1**	**0**	**1**	**0**	**1**
**NSC74859**	**0**	**0**	**10**	**10**	**25**	**25**
GAPDH	16,602	13,164	11,430	11,843	11,541	13,014
PD-L1	12,598	13,043	5,693	3,598	1,579	2,766
Ratio	0.76	0.99	0.50	0.30	0.14	0.21
P-STAT3	15,089	18,865	7,181	7,744	4,896	5,219
Ratio	0.91	1.43	0.63	0.65	0.42	0.40
T-STAT3	15,531	12,367	13,892	13,337	13,722	18,461
Ratio	0.94	0.94	1.22	1.13	1.19	1.42

Figure S16. Uncropped scans with size marker indications of western blots shown in Figure 6H and Figure 6J, and densitometry readings/intensity ratios.

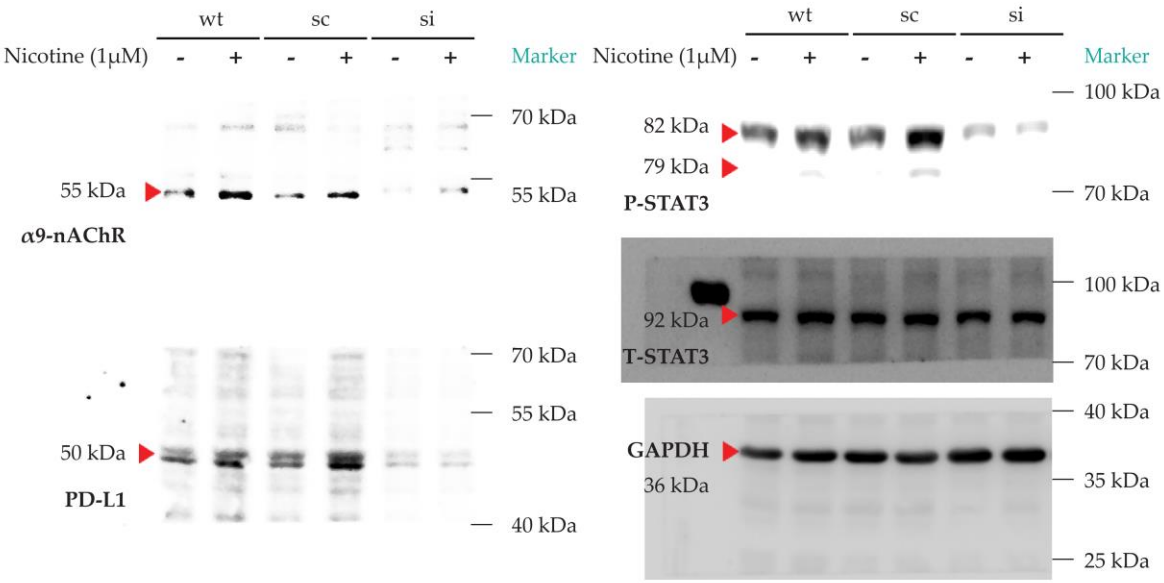

Densitometric values

A2058	wt		sc		si	
**Nicotine (µM)**	**0**	**1**	**0**	**1**	**0**	**1**
GAPDH	13,880	15,871	14,151	13,371	17,932	18,247
α9-nAChR	7,146	16,009	4,481	10,083	2,584	3,254
Ratio	0.51	1.01	0.32	0.75	0.14	0.18
PD-L1	8,887	16,402	10,992	19,563	2,217	1,533
Ratio	0.64	1.03	0.78	1.46	0.12	0.08
P-STAT3	8,893	14,436	9,795	19,517	2,399	1,162
Ratio	0.64	0.91	0.69	1.46	0.13	0.06
T-STAT3	17,780	21,730	18,772	19,406	16,168	15,797
Ratio	1.28	1.37	1.33	1.45	0.90	0.87

Figure S17. Uncropped scans with size marker indications of western blots shown in Figure 6K, and densitometry readings/intensity ratios.

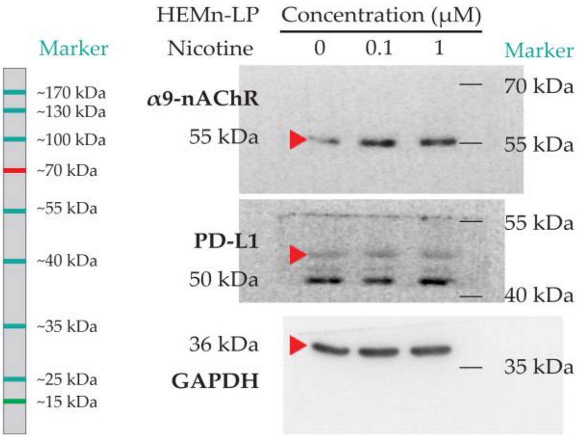

Densitometric values

HEMn-LP	Concentration (µM)		
**Nicotine**	**0**	**0.1**	**1**
GAPDH	35,821	38,681	39,622
α9-nAChR	14,188	34,928	36,227
Ratio	0.40	0.90	0.91
PD-L1	28,355	28,453	31,000
Ratio	0.79	0.74	0.78

Figure S18. Uncropped scans with size marker indications of western blots shown in Figure S5, and densitometry readings/intensity ratios.

© 2019 by the authors. Licensee MDPI, Basel, Switzerland. This article is an open access article distributed under the terms and conditions of the Creative Commons Attribution (CC BY) license (http://creativecommons.org/licenses/by/4.0/).
